# Annotation and detection of drug effects in text for pharmacovigilance

**DOI:** 10.1186/s13321-018-0290-y

**Published:** 2018-08-13

**Authors:** Paul Thompson, Sophia Daikou, Kenju Ueno, Riza Batista-Navarro, Jun’ichi Tsujii, Sophia Ananiadou

**Affiliations:** 1 National Centre for Text Mining, School of Computer Science, Manchester Institute of Biotechnology, University of Manchester, 131 Princess Street, Manchester, M1 7DN UK; 2 Artificial Intelligence Research Center, National Research and Development Agency (AIST), Tokyo Waterfront 2-3-2 Aomi, Koto-ku, Tokyo, 135-0064 Japan

**Keywords:** Pharmacovigilance, Text mining, Corpus annotation, Drug effects, Drug–drug interactions, Adverse drug effects, Resource curation, Events

## Abstract

Pharmacovigilance (PV) databases record the benefits and risks of different drugs, as a means to ensure their safe and effective use. Creating and maintaining such resources can be complex, since a particular medication may have divergent effects in different individuals, due to specific patient characteristics and/or interactions with other drugs being administered. Textual information from various sources can provide important evidence to curators of PV databases about the usage and effects of drug targets in different medical subjects. However, the efficient identification of relevant evidence can be challenging, due to the increasing volume of textual data. Text mining (TM) techniques can support curators by automatically detecting complex information, such as interactions between drugs, diseases and adverse effects. This semantic information supports the quick identification of documents containing *information* of interest (e.g., the different types of patients in which a given adverse drug reaction has been observed to occur). TM tools are typically adapted to different domains by applying machine learning methods to corpora that are manually labelled by domain experts using annotation guidelines to ensure consistency. We present a semantically annotated corpus of 597 MEDLINE abstracts, PHAEDRA, encoding rich information on drug effects and their interactions, whose quality is assured through the use of detailed annotation guidelines and the demonstration of high levels of inter-annotator agreement (e.g., 92.6% F-Score for identifying named entities and 78.4% F-Score for identifying complex events, when relaxed matching criteria are applied). To our knowledge, the corpus is unique in the domain of PV, according to the level of detail of its annotations. To illustrate the utility of the corpus, we have trained TM tools based on its rich labels to recognise drug effects in text automatically. The corpus and annotation guidelines are available at: http://www.nactem.ac.uk/PHAEDRA/.

## Background

Pharmacovigilance [1] (PV) is a vital activity that assesses drugs, in terms of risks and benefits, in order to validate and improve upon their safety and efficacy. Monitoring the effects of drugs is important to determine the effectiveness of treatments in patient populations. In addition, the identification and understanding of adverse drug effects (ADEs) is important for risk assessment. Information about ADEs is also important for stratified medicine, which seeks to identify how drug response may be stratified across patient subgroups [2].

PV is a well-established field, and its research outcomes are used in the creation and update of reference resources that record detailed, evidence-based information about drugs, their effects and interactions (e.g., [3–7]). Since it is estimated that at least 60% of adverse drug reactions are preventable [8], such resources are of critical importance in analysing the appropriateness, effectiveness and safety of prescription medicines. However, maintaining these resources is extremely difficult. Firstly, they are typically manually curated, meaning that they are reliant upon dedicated and intensive efforts of domain experts to carry out extensive surveys of literature and other relevant information sources. Secondly, they can never be considered to be complete, due to the constantly changing evidence about the effects of existing drugs and the development of new drugs, which may be reported in a wealth of different textual resources.

Given the growth of textual data, it is becoming impossible for domain experts to manually curate the information contained within them in an efficient and timely way. Potentially vital information may remain hidden in a deluge of results that are returned when querying these sources. The difficulties in creating and maintaining comprehensive resources are highlighted in a recent survey of several frequently-used drug interaction resources [9], which found several discrepancies amongst the resources, in terms of factors such as the scope of reactions covered, completeness of information about the reactions and consistency of information between the resources. Such inconsistencies could result in patient care being compromised.

In order to mitigate such issues, text mining (TM) techniques have been proven to form the basis for more efficient solutions. They have been used to detect information relevant to drug effects in a range of potentially complementary information sources, including the scientific literature [10–12], electronic health records [13, 14], product labels [15, 16] and social media [17–21], along with pharmacokinetic [22–26] or genetic [27–29] evidence for these effects. TM methods have been used to semi-automate the curation of a number of databases in areas such as biomedicine [30], pharmacogenomics [31] and drug side effects [16], by increasing the efficiency with which articles of interest can be identified and/or by automatically locating relevant details within these articles. Furthermore, TM can be applied to the contents of such databases to uncover meaningful associations amongst drugs [32].

Particularly in the field of biocuration, much attention has been devoted to ensuring that TM methods can be effectively translated into tools that can increase the efficiency of curators’ tasks [30, 33–35]. Additionally, given the variability in curation tasks and working methods, efforts have been made to increase the flexibility with which different TM tools can be integrated within curation workflows in different ways [36–38].

The level of TM-driven support that can be provided to curators for particular tasks is dependent on the sophistication and performance of the tools that are available for a given domain/subject area. The development of such tools is typically reliant on the availability of annotated corpora, i.e., collections of texts manually marked up by domain experts with semantic information pertaining to a domain, which are used for training and evaluating text mining tools.

The levels of semantic annotation in different corpora determine the types of information that can be recognised by TM tools. Named Entities (NEs), i.e., semantically categorised words/phrases, such as drugs and disorders, form the basis for a number of more complex types of annotation. Several efforts have produced corpora annotated with such NEs and/or demonstrated how such corpora can be used to train machine learning (ML) tools to recognise NEs automatically to high degrees of accuracy [39–50].

In many cases, the annotation process involves linking each NE with a unique concept identifier in a domain-specific terminological resource. These resources include the UMLS Metathesaurus [51], the Medical Subject Headings (MeSH) [52], DrugBank [3] and ChEBI [53]. Such linking is important, because of the wide range of ways in which a given concept may be mentioned in text. For example, variant mentions of the disorder concept *dyspnea* may include spelling variations (*dyspnoea*), completely different forms (e.g., *shortness of breath*), abbreviations (e.g., *SOB*) and altered internal structures (e.g., *breath shortness*). Similarly, the medication *etanercept* may be mentioned in various forms, such as a brand name (*Enbrel*), a different form (*Tumor Necrosis Factor Receptor IgG Chimera*), or an abbreviation (*ETN*; *TNFR:Fc*). The NEs in several corpora relevant to PV include manually-assigned concept IDs (e.g., [44, 45, 54, 55]). Such corpora can facilitate the development of *normalisation* methods (e.g., [56–59]), which aim to automatically assign a concept ID in a given terminological resource to each NE. The challenge of this task is that, although terminological resources usually list some synonyms/variant forms for each concept, the range of variants that can actually appear in text is far larger, and mostly unpredictable. However, successful normalisation makes it possible to develop search tools that can automatically locate *all* mentions of a concept of interest in large collections of text, regardless of *how* the concept is mentioned.

Binary relation annotations link together pairs of NEs whose textual contexts connect them in specific ways. The wide range of corpora annotated with binary relations relevant to PV (summarised in Table 1) mainly concern interactions between pairs of drugs (see sentence S1), or different types of relationships between disorders and treatments, e.g., a treatment may improve, worsen or cause a disorder (see sentence S2).

**Table 1:** Summary of corpora annotated with relations relevant to PV

References	Text type	Corpus size	Relation type	No of relations
Gurulingappa et al. [43]	MEDLINE case reports	2972 documents	Adverse drug reaction	6821
Rosario and Hearst [60]	MEDLINE abstracts	100 titles and 40 abstracts	Disorder-treatment	1724
Van Mulligen et al. [61]	MEDLINE abstracts	100 abstracts	Disorder-treatment	668
Uzuner et al. [40]	Clinical discharge summaries/progress notes	477 reports	Disorder-treatment	3462
Roberts et al. [62]	Clinical documents (clinic letters, radiology, and histopathology reports)	150 documents	Disorder-treatment	227
Oronoz et al. [63]	Clinical discharge reports (Spanish)	75 reports	Adverse drug reactions	162
Patki et al. [64]	Social media	10,616 user comments from DailyStrength	Adverse drug reactions	2513
Ginn et al. [65]	Social media	10,822 Tweets	Adverse drug reactions	1436
Li et al. [55]	PubMed articles	1500 articles	Chemical-disorder relations	3116
Boyce et al. [46]	Drug package inserts	64 inserts	Drug–drug interactions	592
Rubrichi and Quaglini [49]	Summaries of product characteristics (Italian)	100 sections on drug-related interactions	Drug–drug interactions	2862
Segura-Bedmar et al. [66]	Free text documents from DrugBank	579 documents	Drug–drug interactions	3160
Herrero-Zazo et al. [50]	Free text documents from DrugBank and MEDLINE abstracts	792 DrugBank documents/233 MEDLINE abstracts	Drug–drug interactions	5028

The number of relations specified in the last column refers only to those of the PV-relevant relation types shown in the column “Relation Type” (in some cases there are other annotated relation types in the corpus)

- (S1) *In vitro interaction of* *prostaglandin F2alpha* *and oxytocin in placental vessels*
- (S2) *Lupus*-*like syndrome* *caused by* *5*-*aminosalicylic acid*

It has been shown that corpora annotated with binary relations can be used to train automated tools to recognise relations in text types with divergent characteristics [46, 67–73], thus allowing the discovery of high-accuracy evidence from multiple information sources that can satisfy a particular information need, e.g., the set of disorders reported to be adversely affected by a given drug.

Despite this, binary relations are limited in terms of the complexity of the information that they can encode. For example, a binary relation representing an adverse drug reaction can only encode the fact that a single drug adversely affects or causes the occurrence of a particular disorder. However, additional information in the text may provide important or even critical details regarding the safe usage of the drug. For instance:

- Adverse reactions may occur not only due to the administration of a single drug, but also according to interactions/combinations of multiple drugs (see sentence S3)
  (S3) *We hypothesize that a pharmacodynamic or pharmacokinetic* *drug interaction between venlafaxine and trimipramine* *involving the CYP2D6 isoenzyme may have played a role in inducing the seizures*.
- The occurrence of a reaction may be restricted to certain types of medical subjects, who may be characterised in various ways, e.g., according to age, gender, disorders suffered at the time of drug administration (see sentence S4)
  (S4) *MM patients* *treated with thalidomide and doxorubicin have a high risk of developing DVT.*
- Different reactions vary in terms of their strength/level of adversity, which may be dependent on patient characteristics (see sentence S5)
  (S5) *We describe a* *life threatening* *side effect of acute epoprostenol infusion (pulmonary edema) in a patient with pulmonary hypertension associated with limited scleroderma and discuss its management and potential etiology.*
- Depending on the type and volume of evidence available, a reaction may be stated with varying degrees of confidence (see sentence S6)
  (S6) *Marked elevation of serum CK may be a* *possible* *complication of olanzapine therapy*.

In this article, we describe the development of a novel annotated corpus, PHAEDRA (PHArmacovigilence Entity DRug Annotation). Uniquely within the field of PV, the 597 abstracts in the corpus include annotations that go beyond binary relations, to encode more complex information, such as the cases exemplified above, in a structured manner. It is intended that PHAEDRA will encourage the development/adaption of machine learning based text mining tools for extracting PV-related information from text, at a level of complexity that has not previously been possible. Ultimately, it is hoped that such tools will lead to the provision of curator-oriented applications that provide sophisticated, efficient and flexible means to explore and pinpoint relevant information in different textual sources, and thus help to increase the coverage, consistency and completeness of information in PV resources.

We encode detailed information about the effects of drugs in PHAEDRA using five different levels of annotation. To ensure the utility of our corpus, all levels of annotation are inspired by, and comparable in scope to, annotation efforts in other domains, for which successful automated recognition has been demonstrated. The five levels are as follows:

1. Three types of NEs that correspond to the important details about drug effects, i.e., drugs, the disorders that they affect and the medical subjects in which the effects occur. We apply an automated normalisation method [59], to link drug and disorder NEs with concept IDs in terminological resources, i.e., MeSH [52] and SNOMED-CT [74], respectively.
2. Four types of complex relation annotations, called *events* [75], which link together an arbitrary number of *participants* (either NEs or other events), to encode detailed information about the behaviour and effects of drugs. Events have previously been used to encode a range of information in texts with divergent characteristics [42, 76–80], and can be recognised automatically by a number of configurable tools [81–92]. Events alleviate several issues with binary relations. For example, our event types *Adverse_Effect (AE)* and *Potential_Therapeutic_Effect (PTE),* which, respectively, encode the harmful and potentially beneficial effects of drugs, identify participants that correspond not only to drugs and the disorders that they affect, but also the medical subjects in which the effects are observed to occur, as in sentence S4. Our *DDI* and *Combination* event types, which encode cases in which multiple drugs are administered together or interact with each other, can themselves act as participants of AE and PTE events. This makes it possible to encode complex causes of drug effects, which involve multiple drugs (as in sentence S3).
3. Three types of *interpretative attributes* assigned to events, which encode whether the events are *negated, speculated* (sentence S6) and their *manner,* i.e., whether the strength, intensity or frequency of the event is *low, high* or *neutral.* This latter information may be relevant in assessing the balance between the risks and benefits of taking particular (combinations of) drugs. For example, the phrase *life*-*threatening* in sentence S5 denotes a severe adverse reaction. In contrast, a *modest* adverse reaction may be considered acceptable, especially if there are other significant benefits to be gained by taking a particular drug/combination. Various studies have annotated and/or developed automated methods to predict similar attributes for events in other domains (e.g., [93–98]).
4. Two types of *static* binary relations between NEs. These are used, as in other event-annotated corpora [76, 99, 100], to encode more detailed information about event participants, and have been shown to improve event extraction results [101]. *Subject_Disorder* relations link medical subjects with their conditions at the time that a treatment is administered. An example of such a relation is shown in sentence S7; structured relationships such as the one shown between *60*-*year old woman* and *diabetes mellitus* would make it possible to explore, e.g., the range of disorders suffered by patients who have such an adverse reaction to *gliclazide.* Relations of type *is_equivalent* link together different names for the same concept (e.g., full name and acronym/abbreviation, brand name and common name for a drug). These relations can supplement our automatically-added associations between NEs and concept IDs in allowing links to be established between events that refer to a common concept in different ways.
  (S7) *A* *60*-*year*-*old woman* *with* *diabetes mellitus* *developed an acute icteric hepatitis*-*like illness 6* *weeks after the initiation of gliclazide therapy.*
5. Co-reference annotations between anaphoric event participants and their co-referent NEs in nearby sentences. Our annotation scheme requires that all participants of a given event occur within a single sentence. However, some participants may correspond to anaphoric expressions, such as *it, the drug, this disease,* etc., referring to NEs introduced earlier in the text. We allow event participants corresponding to anaphoric expressions to be annotated, but require such expressions to be linked to their co-referent NEs in other sentences, to allow events to be correctly interpreted [81]. Co-reference annotation efforts in other biomedical corpora [102, 103] have been used to demonstrate the feasibility of developing novel approaches to co-reference resolution [104–106].

The creation of PHAEDRA was driven by detailed annotation guidelines, developed in consultation with domain experts, and used to ensure consistent annotation. Such consistency is quantitatively demonstrated by generally high levels of inter-annotator agreement, reaching as high as 92.6% F-Score for identification of named entities and 78.4% F-Score for the identification of complex events, when relaxed matching criteria are applied. The potential utility of PHAEDRA for developing/adapting text mining tools is shown through our training of baseline tools for NE and event recognition; encouraging levels of performance are illustrated, which compare favourably to other related efforts.

## Methods

In this section, we report on the design of the PHAEDRA corpus. We firstly describe the methodology that we used to collect documents for inclusion in PHAEDRA. Subsequently, we provide further details about each of the five different annotation levels. Finally, we explain our approach to normalising NE annotations to concept IDs in appropriate terminological resources.

### Document selection

PHAEDRA is comprised of documents that are gathered based on: (1) publicly available resources (relevant to the PV domain) and (2) our own search—conducted in October 2015—for more recently published scientific abstracts. Specifically, we made use of the publicly available ADE [43], DDI 2013 [50] and PK [24] corpora, which all contain annotations pertaining to PV-relevant named entity types. In searching for further documents, two strategies were taken. Firstly, we took the “Top 100 drugs” listed in the online pharmaceutical encyclopaedia Drugs.com and compiled a list of PubMed titles referenced in the “Side Effects” section for each drug. The PubMed eUtilities [107] were then used to download the content of corresponding abstracts. We also explored PubMed further by specifying the following query: *(hasabstract[text] AND adverse drug reactions[MeSH Terms] AND drug interactions[MeSH Terms] AND (“2005/01/01”[Date* - *Publication] : “2016”[Date* - *Publication]) AND English[lang])*.

Overall, 2968 abstracts were gathered. In order to select the subset that would form our corpus, the abstracts were ranked according to the number of unique drug names that they contain. To this end, a drug NE recognition tool [108] was employed to annotate drug names in the set of otherwise un-annotated abstracts (retrieved based on information from Drugs.com and by searching PubMed). By selecting the 600 documents containing the highest number of drug names, we finally formed a corpus comprising 227, 125 and 52 abstracts from the ADE, PK and DDI 2013 corpora, respectively, and 193 abstracts from our own search.

### Annotation levels

The design of each annotation level was guided by an examination of a wide range of relevant MEDLINE abstracts, to determine the types of information that are most frequently specified, combined with discussions with domain experts, which helped us to focus on the most important types of information to be encoded in our scheme.

### Named entity annotation

To our knowledge, PHAEDRA is the first freely available corpus focussing on PV that simultaneously includes annotations corresponding to all of our three chosen NE types, as a basis for linking the effects of drugs with information about the medical subjects in which they occur. The scope of each NE type is outlined in Table 2.

**Table 2:** NE types

Entity type	Description	Examples	
Pharmacological_substance	Pharmacological substance that may or may not be approved for human use	Genes/gene products used as therapeutic agents	*Echistatin*
		Generic drug names	*Didanosine*
		IUPAC and IUPAC-like chemical names of drugs	*5-hydroxy-* l *-tryptophan*
		Endogenous substances administered as exogenous drugs	*Insulin*
		Toxins	*1-methyl-4-phenyl-1,2,3,4-tetrahydropyridine*
		Excipients	*Isopropyl myristate*
		Generic or chemical names of metabolites	*Threohydrobupropion*
		Drug brand names	*DIAMOX*
		Names of groups of drugs	*Fluoroquinolones*
		Expressions characterising general classes of drugs	*Dopamine D1 receptor antagonist*
Disorder	Observation about a medical subject’s body or mind that is considered to be abnormal or caused by a disease, pharmacological substance or DDI	Medical conditions	*Pulmonary embolism*
		Abnormality in physiological function	*Hyperlocomotion*
		Pathological process	*Fibrosis*
		Neoplastic process	*Intestinal adenocarcinoma*s
		Damage caused by disease or drugs	*Cerebellar damage*
		Mental or behavioural issue	*Drug abuse*
		Injury or poisoning	*Clinical toxicities*
		Viruses/bacteria	*Micrococcus luteus*
		Sign or symptom	*Nausea*
		Abnormality in clinical attributes or measurements	*Increased urine sodium*
Subject	An organism, cell line, bacterium or group thereof, whose characteristics are under discussion. The organism may be human or otherwise	General references to groups of subjects	*Children*
		Names of specific species under discussion	*Mice*
		Names of bacteria under discussion	*Klebsiella oxytoca*
		Expressions that specify a number of subjects	*16 patients*
		Descriptions of subject characterstics	*50-year old male patient*

We use the *Pharmacological_substance* label, since this category is largely based on the one used in the DDI corpus [50], whose scope is wider than prescription drugs. The *Disorder* category is based on, but not restricted to, the UMLS [51] *Disorder* semantic group. Our annotators were encouraged to consult the Medical Dictionary for Regulatory Activities (MedDRA) [109] as a guide to helping to determine when a phrase should be considered as a disorder. Most *Subject* phrases have a similar scope to [42], although our annotations extend beyond human subjects to cover different organisms and their sub-structures.

**Fig. 1:**
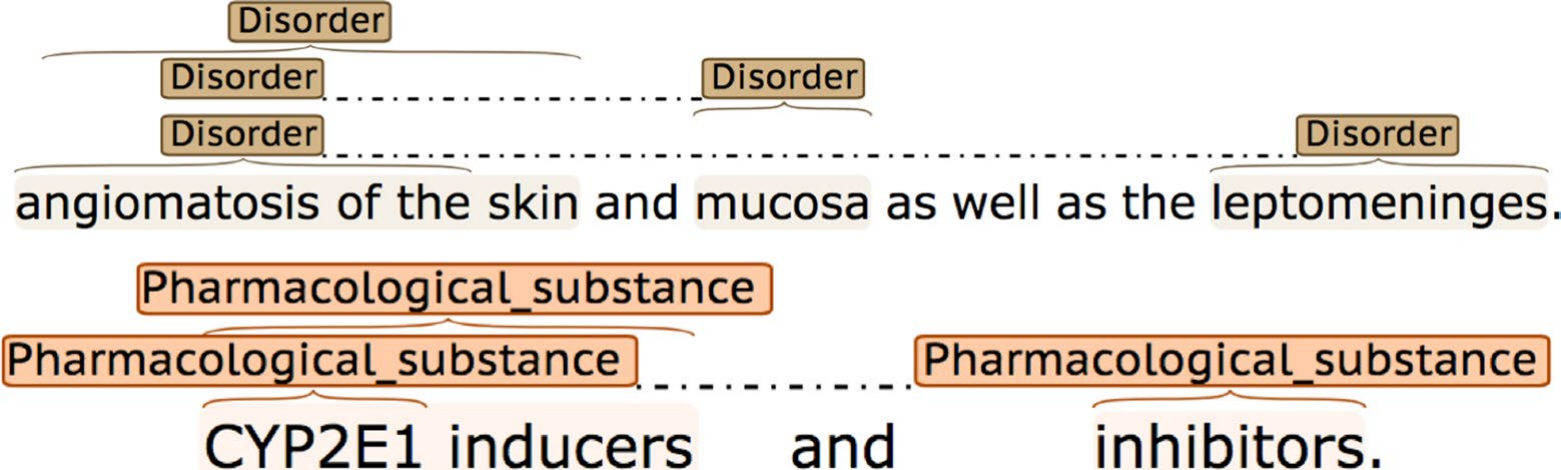
Discontinuous NE annotations

Annotated NE spans may either be *continuous* or *discontinuous.* Discontinuous annotations consist of two or more disconnected spans of text, which denote a complete NE when concatenated. Discontinuous annotations are most often used in coordinated phrases, in which multiple NEs are mentioned, but there is a “shared” part of the NE that is mentioned only once. Examples are shown in Fig. 1.

### Event annotation

**Fig. 2:**
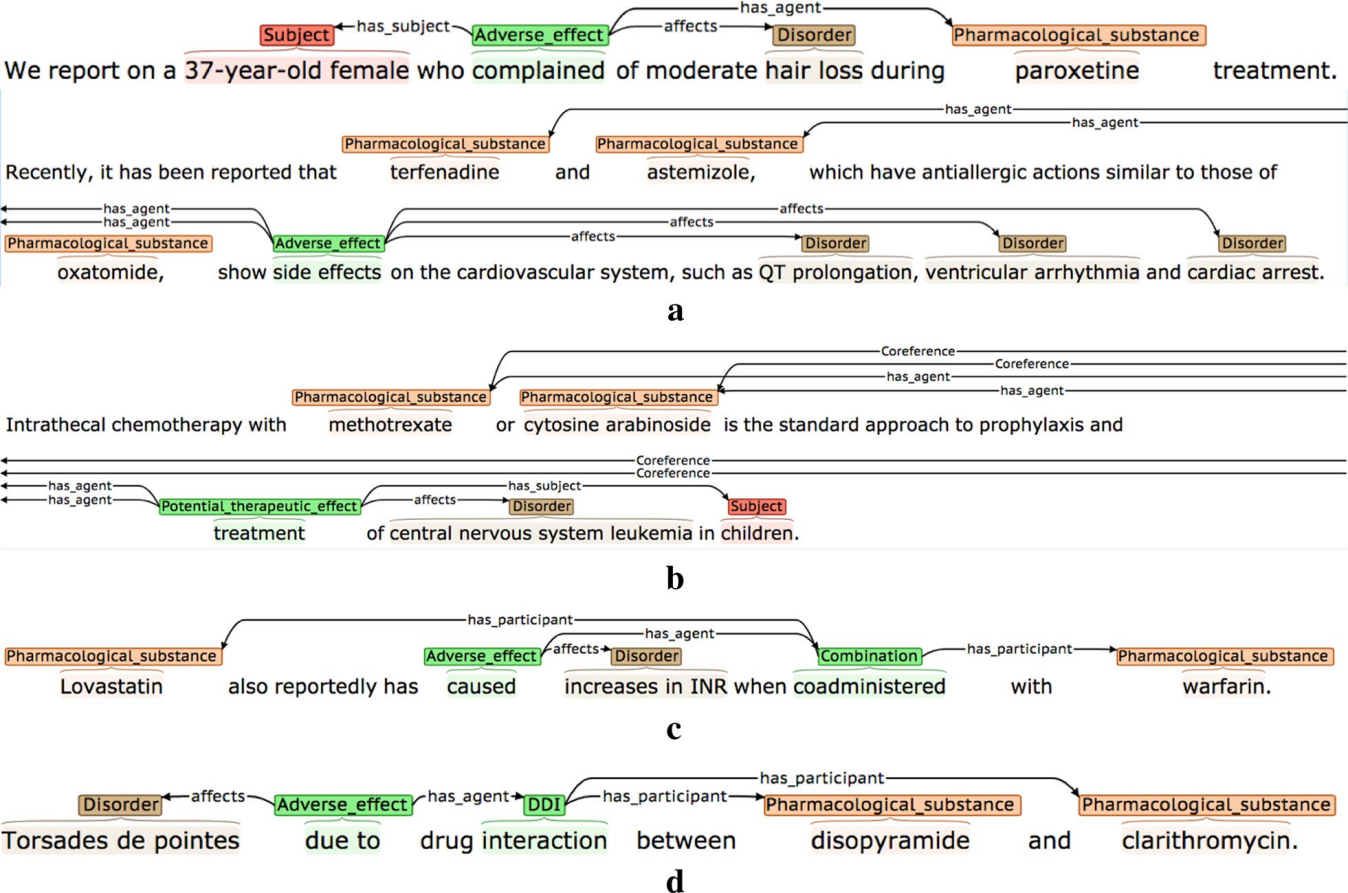
Examples of different event types. The semantic types of the events are **a** Adverse_effect, **b** Adverse_effect, **c** Combination, and **d** DDI

Definitions and possible participants of the four event types are shown in Table 3, while examples of each are shown in Fig. 2. Each event annotation is *anchored* to a word or phrase (known as the *trigger)* that denotes the occurrence of the event in the sentence. As can be seen in the examples in Fig. 2, the triggers of *AE* events often denote causality, using words and phrases like *caused* or *due to,* while *PTE* triggers frequently correspond to words or phrases denoting treatments or drug administration. Each event participant is assigned a *semantic role* label to characterise its contribution towards the event description, and is linked to the trigger. Following most other event annotated corpora, and according to the current capabilities of available automated event extraction tools (e.g., [89, 91, 92]), our scheme requires that event participants occur within the same sentence as the trigger. Different event types use different sets of semantic roles according to their semantics. The semantic roles that we have defined for each event type (e.g., *has_agent*) are outlined in the *Possible participants* column of Table 2 and are exemplified in Fig. 2, where the semantic roles are shown as labels on the arcs which link the participants to the trigger.

**Table 3:** Event types

Event type	Definition	Possible participants
Adverse_effect (AE)	A pharmacological substance or combination/interaction between pharmacological substances has an effect on the body that is considered to be undesirable. More specifically, the substance, combination or interaction causes a disorder to manifest itself, or to become worse	*has_agent*: Pharmacological substance or combination/interaction of substances responsible for the adverse effect *affects*: Disorder resulting from or worsened by administration of the Agent *has_subject*: The individual or group in which the drug effect is specified to occur
Potential_therapeutic_effect (PTE)	A pharmacological substance or combination/interaction of pharmacological substances is being administered, with the intention of having a therapeutic effect	*has_agent*: Pharmacological substance or combination/interaction of substances responsible for the therapeutic effect *affects*: Disorder improved or cured by administration of the Agent *has_subject*: Individual or group in which the therapeutic effect is specified to occur
Combination	A specification that two or more pharmacological substances are being used at the same time (e.g., they have been co-administered)	*has_participant*: A pharmacological substance being combined/co-administered
DDI	A specific mention that there is an interaction between two or more pharmacological substances	*has_participant*: A pharmacological substance involved in the interaction *has_subject*: Individual or group in which the DDI is specified to occur

For each event type, certain participant types are obligatory (i.e., the participant, or an anaphoric expression referring to the participant, *must* be present in the same sentence as the trigger in order for the event to be annotated), while other participant types are optional; the restrictions are detailed in Table 4. For each event type, it is permitted to annotate multiple participants with the same role label.

**Table 4:** Conditions for event annotation

Event type	Conditions
Adverse_Effect (AE)	At least ONE *has_agent* participant
Potential_Therapeutic_Effect (PTE)	At least ONE *has_agent* participant At least ONE *affects* participant OR at least ONE *has_subject* participant
DDI	At least TWO *has_participant* participants
Combination	At least TWO *has_participant* participants

The *has_agent* participant for both *AE* and *PTE* event types may either be a simple drug NE, or a more complex cause, encoded by a *DDI* or *Combination* event. The *DDI* event type is annotated when an interaction is explicitly mentioned, while the *Combination* type is used in cases where it is simply stated that two or more drugs are administered together. The *Combination* event type makes it possible to differentiate between cases where a number of different drugs taken in isolation are listed as common causes of a given adverse effect (see second *AE* event example in Fig. 2), and cases where the *AE* is only observable when two or more drugs are co-administered (see example for *Combination* event in Fig. 2). To collect the maximum amount of evidence about the range of ways in which all event types can be described in text, all instances of *DDI* and *Combination* events are annotated, regardless of whether they occur as the *has_agent* participant of *AE* and *PTE* events.

For *AE* events, it may be useful to detect statements that a given medication has a potentially harmful effect, even if it is not stated which disorder(s) are affected. This additional information may be present elsewhere in the abstract, or else such underspecified information may help to flag drugs for which further research is needed. Although *PTE* events can be considered to be the opposite of *AE* events, we impose a different set of restrictions. Since most pharmacological substances are mentioned in the context of treatments, a simple statement that a drug has been administered is not particularly informative, and hence is not annotated as an event. Rather, we require the presence of one of two types of additional participants. These additional participants can correspond either to one or more *affects* participants, in order to allow the collection of evidence about which disorders are positively affected by which treatments, or to one or more *has_subject* participants, in order to collect evidence about the types of patients in which a treatment can be used safely. For *DDI* and *Combination* events, at least two drugs must be identified as participants for the events to make sense.

### Event attributes

**Fig. 3:**
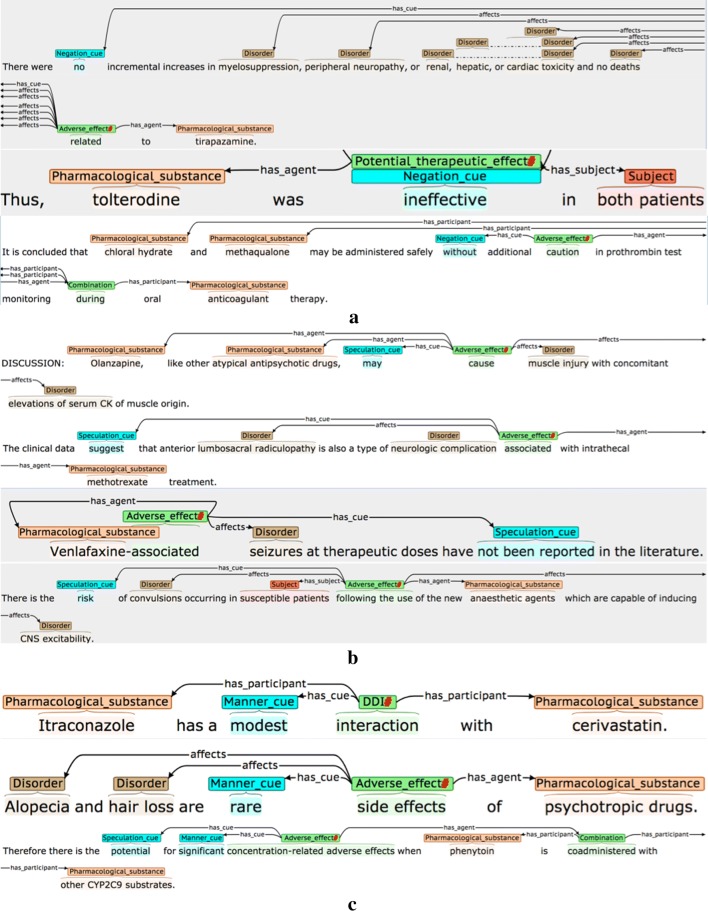
Event attribute annotation examples. The type of attribute being illustrated are **a** Negated, **b** Speculated and **c** Manner. Red hash (#) characters denote events that are assigned one or more non-default attribute values

Table 5 details the three interpretative attributes that are assigned to each event, while Fig. 3 provides some examples. Where possible, attribute-specific *cue phrases* are annotated and linked to the event. These are words/phrases in the same sentence as the event, which are used by the annotator to determine the chosen (non-default) value for a given attribute. It has been shown [95] that the annotation of such clue words can increase the accuracy of tools that are trained to assign the values of the attributes automatically.

**Table 5:** Event attributes

Attribute	Description	Possible values
Negated	Denotes whether or not there is explicit evidence in the sentence that the event should be negated	*True*: Explicit negation evidence is present *False*: There is no evidence that the event should be negated (default value)
Speculated	Denotes whether there is some degree of uncertainty or speculation as to whether the event will actually take place	*True*: There is explicit mention that: • there is uncertainty about whether the event will actually take place • there is a risk that the event will take place • the event may not take place all of the time • there is a lack of evidence/knowledge about the truth of the event *False*: There is no evidence of any of the above (default value)
Manner	Denotes whether the manner of the event (i.e., the rate, intensity, strength or level of significance of the event) is higher or lower than would be expected by default	*Low*: There is explicit indication that the manner of the event is lower than would be expected by default, e.g., it happens with low intensity, happens rarely or is not considered to be significant *High*: There is explicit indication that the manner of the event is higher than would be expected by default, e.g., it happens with high intensity, it happens very frequently, or is considered to be very significant *Neutral*: There is no explicit indication that the manner of the event is higher or lower than would be expected by default (default value)

The second example of a negated event in Fig. 3 shows how some words may simultaneously act as event triggers and negation cues. Here, the word *ineffective* denotes that there is NO therapeutic effect, and hence it encodes a negated PTE. The final example for *Manner* in Fig. 3 illustrates how multiple types of interpretative information may be specified in the context of events; the significant effects of the stated drug combination may not happen in all patients, and hence the AE event is assigned a *Manner* value of *High,* as well as being marked as *Speculated.*

### Relation annotation

**Fig. 4:**
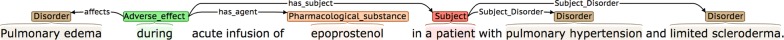
Example of a *Subject_Disorder* relation

The first of our two static binary relation types (*Subject_Disorder*) connects *Subject* phrases and *Disorders*, when the mentioned disorder corresponds to a complaint suffered by the subject(s) at the time when pharmacological substances are administered. We consider such relations to be important, since, in the context of discussing drug effects, medical subjects are frequently characterised by their existing medical conditions. Figure 4 shows an example of such a *Subject_Disorder* relation.

In Fig. 4, the text span *a patient with pulmonary hypertension and limited scleroderma* could feasibly be annotated as a continuous *Subject* phrase. However, this would “hide” specific information about the characteristics of the patient, i.e., the particular disorders from which they suffer. The automatic recognition of such long spans is also likely to cause problems for an ML-based NE recognition system. Hence, we annotate *Subject* and *Disorder* phrases separately, and link them via *Subject_Disorder* relations.

**Fig. 5:**
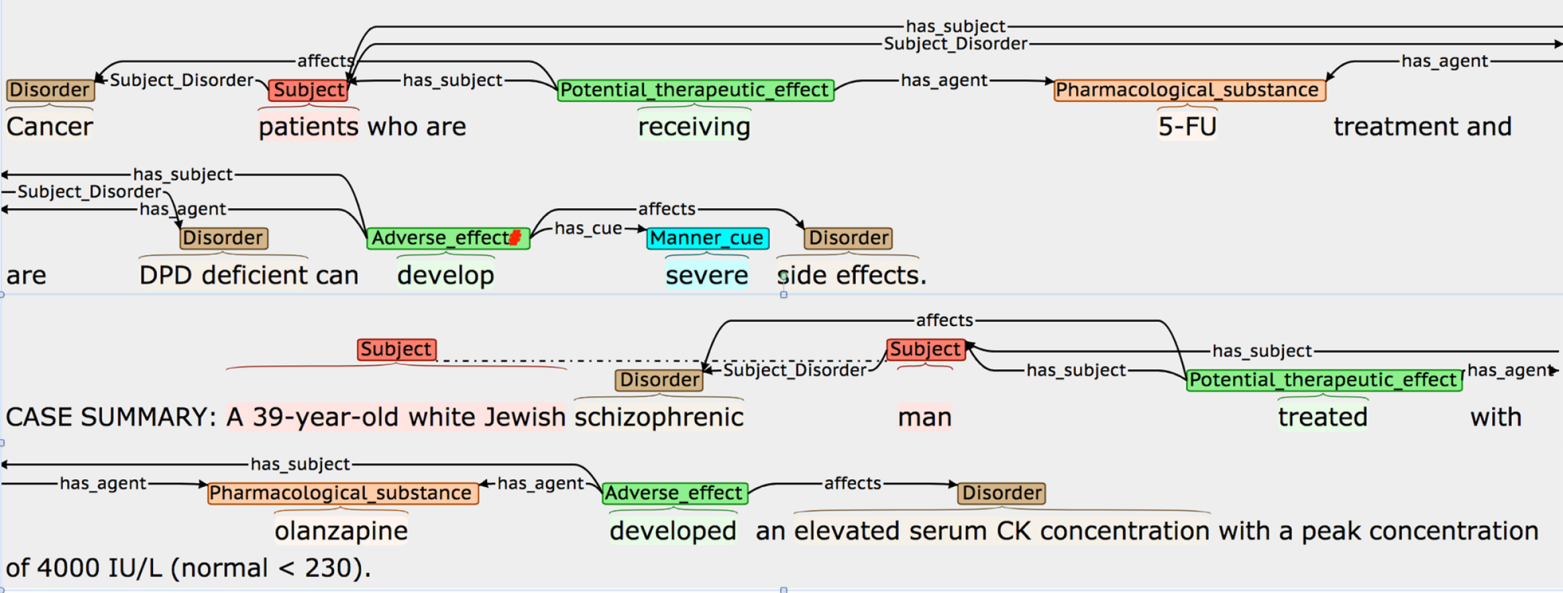
More complex *Subject_Disorder* examples

Figure 5 illustrates some of the diverse ways in which *Subject_Disorder* relations can be described in text. In the first sentence, the disorders suffered by patients are interspersed with the drug administered. In the second sentence, the condition suffered by the patient occurs as an integral part of a phrase describing their other characteristics, which requires the use of a discontinuous *Subject* annotation to allow the disorder to be identified separately.

**Fig. 6:**
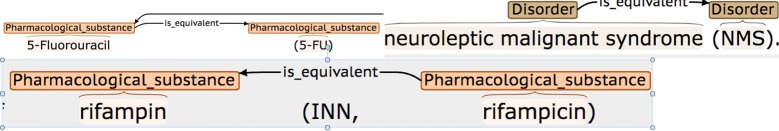
Examples of *is_equivalent* relations

Our second binary relation type, *is_equivalent,* allows links to be established between NEs that constitute alternative names for the same concept within the same sentence. Equivalences may correspond to full drug names/disorders and their abbreviations, to generic drug names and their corresponding brand names or synonyms, etc. Some examples are shown in Fig. 6. Similar equivalences have previously been annotated in the context of biomolecular events [81]. Since alternative names for a concept may be used in different parts of a document, the detection of *is_equivalent* relations could help to establish groups of events that mention a common participant, even if different words or phrases are used to refer to the same participant. These relations complement the information provided through the application of applying our automated normalisation method to NEs, described below.

### Co-reference annotation

Our annotation scheme requires that event triggers and participants in PHAEDRA must occur within the boundaries of a single sentence. This helps to ensure that event annotation is feasible for annotators, whilst also allowing existing event extraction tools to be applied to the corpus. However, resolving underspecified expressions that act as participants to their co-referent NEs in other sentences is an important step to ensure the accurate extraction and/or interpretation of events.

**Fig. 7:**
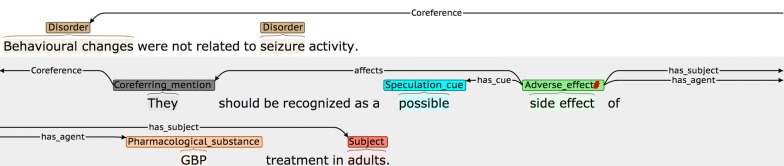
Co-reference annotation example

Given that our main focus is on events, and the fact that annotators have to perform multiple levels of annotation, our coreference annotation does not aim to cover such a wide scope as efforts such as [103], which annotated all coreferring base noun phrases. Rather, our coreference annotation is restricted to linking event participants that are underspecified phrases to their antecedent NEs, as follows (an example is shown in Fig. 7):

- If the *only* reference to an event participant within an event-containing sentence is an underspecified expression that is too general to be annotated as an NE (e.g., *it, this drug, such disorders,* etc.), then the expression is annotated using the special category *Coreferring_mention,* as long as BOTH of the following hold:
  - There is an annotated NE in a nearby sentence to which the underspecified expression refers.
- Linking the *Coreferring_mention* as a participant of the event results in an event that is valid, according to the restrictions set out in Table 4.
- The *Coreferring_mention* annotation and the previously annotated NE are linked using a *Coreference* relation.

### Normalisation of NEs

We have applied an automatic normalisation method to assign concept IDs to two of our three NE types, i.e., *Pharmacological_substance* and *Disorder*. Both of these concept types are extensively covered in existing terminological resources, and their normalisation has been demonstrated to be feasible by various previous studies (e.g., [54, 55, 110, 111]). Although some terminological resources additionally include concepts that correspond to characteristics of *Subject* annotations (e.g., *Patient or Disabled Group* and *Population Group* in the UMLS Metathesarus), *Subject* annotations are less straightforward to map to specific concepts, according to the fact that the characteristics of medical subjects are highly divergent, and their descriptions may include multiple types of information, including number, gender, age, ethnicity, occupation etc.

To carry out mapping of *Pharmacological_substance* and *Disorder* annotations in PHAEDRA to concept IDs in terminological resources, we considered two previously developed normalisation methods. The first [112] is based on the use of *string similarity* (according to the Jaro-Winkler distance [113]) such that, if an NE does not match exactly to a concept variant listed in the resource, the NE is mapped to the variant with which it bears with the greatest level of similarity. The second method, HYPHEN [59], takes a different approach, in that it is a *hybrid* method that employs a pipeline of different techniques to *generate* variations of the original NE mention (based on systematic syntactic and semantic variations of the original mention) and tries to match these generated variants against existing variants listed in the target terminological resources.

To determine which of the above methods we would employ for concept normalisation in PHAEDRA, we compared their performance on a pertinent task, i.e., normalisation of disease mentions in the test partition of BioCreative V CDR corpus to MeSH concept IDs [55]. The results are shown in Table 6.

**Table 6:** Comparison of normalisation method performance on disease normalisation of gold standard diseases in test partition of the Biocreative V CDR corpus

Method	Precision	Recall	F-score
String similarity-based [112]	89.51	81.94	85.56
Hybrid syntactic/semantic techniques (HYPHEN) [59]	92.03	82.55	87.03

Given that HYPHEN achieved superior results in the common evaluation task, we adopted it to normalise the NEs in PHAEDRA. HYPHEN employs the following six individual techniques (see [59] for further details):,

1. Acronym and abbreviation expansion and context-sensitive disambiguation (e.g., *elevated ICP* -> *elevated intracranial pressure*).
2. Conversion of plural forms to singular (e.g., *thrombi*->* thrombus*).
3. Generation of English equivalents of Neoclassical compounds (e.g., *hyperglycemia* -> *high blood sugar)*.
4. Generation of Neoclassical equivalents of English terms (e.g*., thyroid englargement* -> *thyromegaly)*.
5. Generation of syntactic variants (e.g., *abdomen pain* -> *painful abdomen*).
6. Generation of synonyms (e.g., *cardiac asystole* -> *cardiac arrest*).

The processes are applied in the order shown above, based on the results of experiments to determine the optimal ordering. The output of each process is passed as input to the next technique in the sequence. This can increase the accuracy of normalisation, since multiple transformations are sometimes necessary to allow mapping to the terminological resource, e.g., *hypertensive eyes* -> (singular) -> *hypertensive eye* -> (syntactic) -> *eye hypertension* -> (Neoclassical) -> *ocular hypertension* [*UMLS: C0028840*]. The pipeline is terminated as soon as one of the techniques generates a variant that matches a term in the terminological resource.

## Results and discussion

In this section, we firstly cover several statistics regarding the PHAEDRA corpus. Initially, we present and discuss inter-annotator agreement (IAA) rates between two annotators for different levels of annotation in a subset of the corpus, as a means of determining the quality of the annotations. We subsequently report on various statistics regarding the annotations in the full corpus, including the results of applying the HYPHEN method to normalise the *Disorder* and *Pharmacological_substance* NE types, and we highlight some of the annotation trends, which help to justify that choices made in the design of the corpus were well motivated. Finally, we describe our experiments in using the corpus to train ML classifiers to recognise two levels of information automatically, i.e., NEs and events.

### Ensuring annotation quality

We trained two annotators with domain expertise in the application of the annotation scheme and the use of the *brat* annotation tool [114]. Several rounds of practice annotations resulted in updates being made to the guidelines, to resolve potential weaknesses and omissions in the initial version. All 597 abstracts were annotated by a single annotator; one quarter (i.e., 150) of the same set of abstracts was independently annotated by the second annotator at a mid-point in the annotation effort, to allow IAA results to be calculated and analysed. As a result, the guidelines were further revised; the main annotator re-reviewed their previous annotations and completed the annotation of the remaining abstracts. In this section, we report on the IAA rates for the different levels of annotation, and discuss some of the more challenging cases.

### NE agreement rates

Table 7 reports the IAA rates for NEs, with separate figures for *exact* span agreement (i.e., both annotators have selected exactly the same NE category *and* the same text span) and *relaxed* span agreement (i.e., the annotators have selected the same NE category and *overlapping* text spans). Although consistent span selection can be important for ML algorithms, the potential variability of certain NEs can make many span selection choices difficult, despite the provision of numerous pointers in the guidelines. Therefore, overlapping spans provide evidence that the same NE has been identified by both annotators, even if the spans do not match exactly.

**Table 7:** NE agreement rates (F-Score)

Category	Relaxed agreement (%)	Exact agreement (%)
Pharmacological_substance	96.0	92.8
Disorder	91.9	80.7
Subject	81.1	81.1
TOTAL	92.6	86.0

The overall relaxed and exact agreement rates are very high, and show that the annotators have a similar understanding of which phrases should be annotated as NEs. The results are comparable with previous efforts that annotated similar entity types [42, 61, 62]. Only the *Disorder* category exhibits a notable discrepancy between exact and relaxed matching. Some examples of disagreements for *Disorder* spans are shown in Table 8.

**Table 8:** Disagreement between annotators for *Disorder* spans

Annotator 1 span	Annotator 2 span
*pulmonary sarcoidosis at stage II*	*pulmonary sarcoidosis*
*nodular infiltrations in the lung parenchyma*	*nodular infiltrations*
*scleroderma*	*limited scleroderma*
*nonfatal myocardial infarction*	*myocardial infarction*
*hepatic failure*	*fulminant hepatic failure*
*overt upper gastrointestinal bleeding*	*upper gastrointestinal bleeding*

Descriptions of disorders often include reference to anatomical entities and/or adjectives describing the nature of the disorder, which may extend beyond a single noun phrase into a following prepositional phrase. According to the guidelines, such types of information should only be included within *Disorder* spans if they are considered to be part of the disorder name. This can be a difficult choice, especially if the disorder name is not present in the MedDRA resource used as a guide by the annotators, as is the case for *limited scleroderma.* The word *limited* appears to refer to the extent of the disorder, and hence, it would be feasible to consider it as a cue for *Manner.* However, the complete span *limited scleroderma* is listed elsewhere as a named subtype of *scleroderma* [115].

Agreement rates for *Subject* annotations are the lowest of the three NE categories, although there is no discernible difference between the exact and relaxed matching rates. Disagreements generally occurred when patients were characterised in less usual ways. Examples include *elderly residents of a long*-*term care facility, patients undergoing parathyroidectomy* and *patients for whom surgery is not possible.*

### Event agreement rates

We take a similar approach to calculating IAA for events to that introduced in [79], by determining two types of agreement rates that correspond to the different stages of event annotation:

1. Event identification (i.e., the extent to which the annotators agree that particular events are described in a given sentence).
2. Participant identification (i.e., the extent to which the annotators agree on which participants to annotate for events whose existence they agree upon).

Event identification involves selecting an appropriate trigger word or phrase for the event. In [79], agreement for this task was assessed by determining the extent to which annotators selected the same (or overlapping) trigger spans. However, the event types of interest in that corpus (i.e., gene regulation and expression events) were rather semantically restricted, as evidenced by the fact that around 50% of the 3000 events in the corpus were described using one of ten commonly occurring trigger words.

We found that the diversity of possible triggers for our event types was much greater, especially for AE and PTE events. For example, while the most preferable triggers for AE events are those denoting causality or association, e.g., *produce* or *associated,* there are many cases in which a sentence describing an AE does not include such a word. Thus, the guidelines suggest possible alternatives, including temporal-related words (e.g., *after*) conveying that an adverse effect occurred *following* the administration of a drug, or else a word denoting that the drug is harmful (e.g., *toxicity*), etc.

Given the potential difficulty in selecting an appropriate event trigger, and the fact that there may be multiple possible candidates in a sentence, we have defined both *strict* and *relaxed* matching criteria for evaluating event identification agreement. The *strict* criterion requires that the event trigger spans chosen by both annotators include some degree of overlap, while the *relaxed* criterion aims to accurately pair triggers that do not necessarily overlap, by assuming that they refer to the same event if any of the following conditions hold:

- Triggers overlap AND they are assigned the same event type; OR
- Triggers occur within 20 characters of each other AND they are assigned the same event type; OR
- Triggers occur in the same sentence AND they are assigned the same event type AND they share at least one “core” participant (i.e., at least one *has_agent, affects* or *has_participant* participant must be the same in the matched events).

Table 9 shows agreement rates for event identification, which are closely comparable to those achieved for the gene regulation and expression event annotation task reported in [79], where agreement levels of around 77% F-score were achieved.

**Table 9:** Event identification agreement rates (F-score)

	Strict agreement (%)	Relaxed agreement (%)
Combination	65.3	74.9
DDI	86.5	87.1
PTE	60.2	72.5
AE	63.9	80.3
Total	65.0	78.4

The high agreement rates for DDI events, with little difference between strict and relaxed matching, are to be expected, since such events are almost always denoted by the word *interaction* or *interactions.* Whilst *Combination* events have the same structure as DDI events, the lower agreement is likely to be explained by the greater variety in possible triggers for *Combination* events, together with the potential ambiguity of some of these triggers. For example, although the word *and* is frequently a trigger for *Combination* events when it is used to conjoin two pharmaceutical substances, there are cases where *and* does not refer to their combined administration, and so careful reading may be required. Cases such as the one shown in sentence S8, where there is disagreement between the annotators about whether the trigger should be *both* or *and,* help to explain why there are discrepancies between the strict and relaxed matching rates.

- *(S8) We discuss a case with significant progressive peripheral neurological deterioration following administration of* *both* *fludarabine* *and* *cytarabin.*

**Fig. 8:**
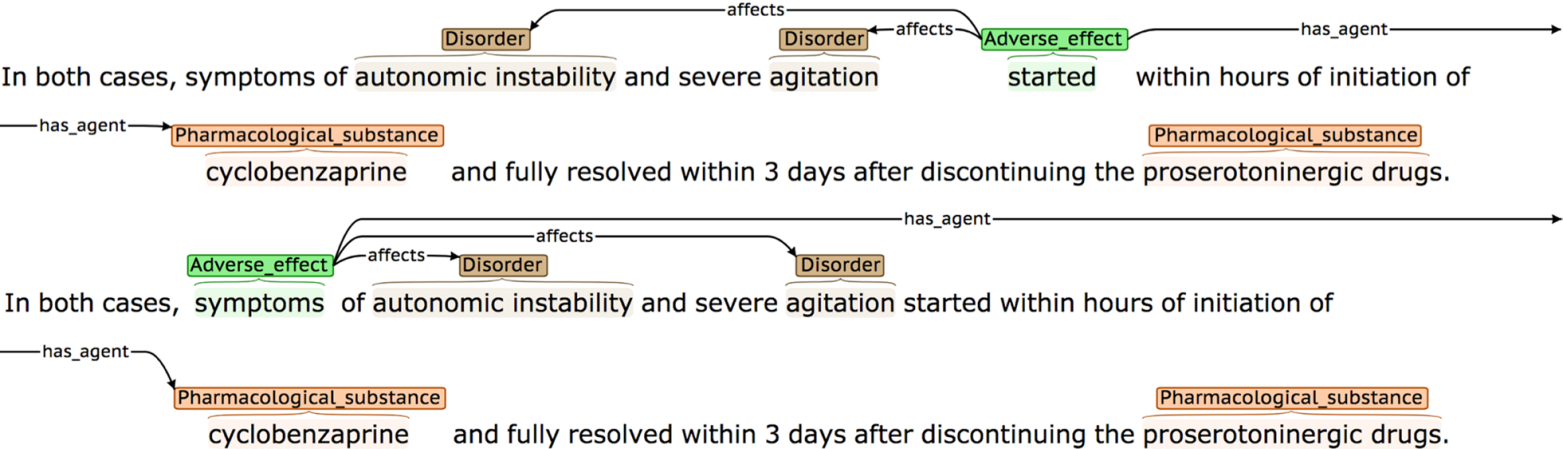
Example of disagreement about event triggers

For AE and PTE events, there is greater discrepancy in the triggers chosen by each annotator. Figure 8 illustrates an example of such a discrepancy with an AE event. Since there are no preferred trigger types in this sentence, i.e., words corresponding to causation, association or risk, one of the alternative guidelines for trigger selection must be followed. Each of the annotators used a different guideline, which resulted in the selection of different triggers:

- A word denoting that the disorder occurred whilst the a substance was being taken (*started*)
- A word conveying the harmful effects of the substance (*symptoms*)

In Table 10, we show the extent to which the annotators identified the same participants with the same semantic roles for events whose existence they agreed upon (according to the relaxed criteria introduced above). Event participants chosen by the two annotators are considered to match if their text spans overlap, and they are assigned the same semantic role.

**Table 10:** Event participant agreement rates (F-score)

Role	Agreement (%)
has_participant	96.1
has_agent	86.2
Affects	90.1
has_subject	73.0
Total	88.2

The levels of agreement for participant identification and classification reach similar levels to those reported in [79] (i.e., 88% F-Score). Disagreements concerning *has_agent* are more frequent than for *affects* participants. The most common type of disagreements arising in relation to this role concern whether the agent corresponds to a single pharmacological substance or a combination of them. For example, in sentence (S9), a careful reading is required to understand that the therapy for Parkinson’s disease is a combination of *ropinirole, levodopa* and *carbidopa,* and hence the *has_agent* participant of the PTE event in this sentence should be *Combination* event, which links them together.

- *(S9)* *Ropinirole* *was added to his current therapy for Parkinson disease, with a corresponding decrease in the dose of* *levodopa/carbidopa* *to allow levodopa sparing.*

**Fig. 9:**
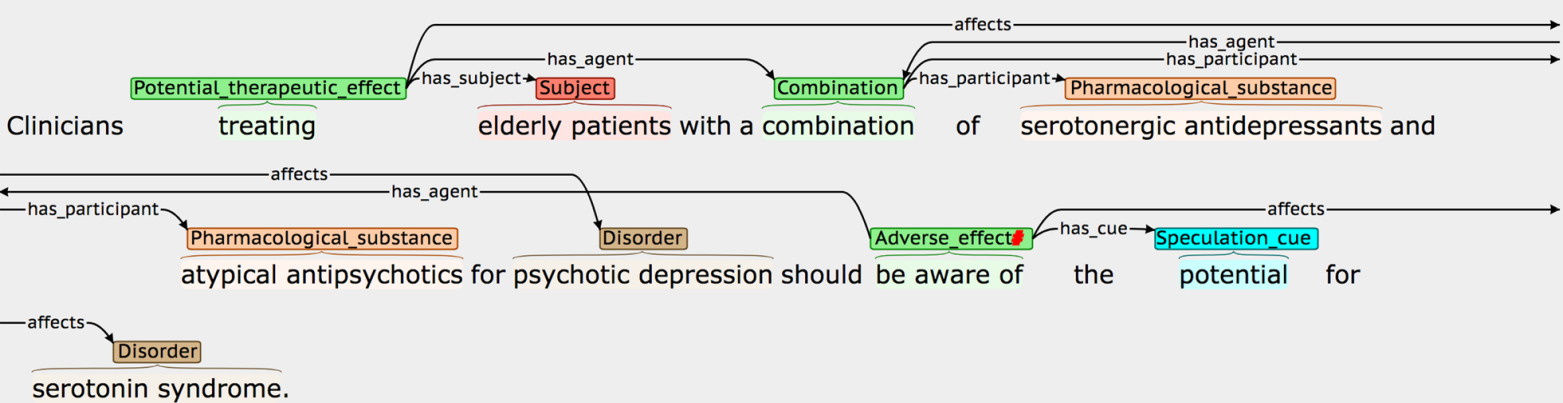
Example of missed *has_subject* participant for AE event

We found that *has_subject* participants are sometimes missed when there are multiple events in a sentence and the subject information is not close to the event trigger. In the sentence shown in Fig. 9, for example, one annotator failed to link the *Subject* phrase to the AE event as well as the PTE.

### Relation agreement rates

Table 11 shows the agreement rates for our two relation types. These figures are comparable to those reported for a number of different medically-related relation annotation tasks (e.g., [61, 62]).

**Table 11:** Relation agreement rates (F-score)

Relation type	Agreement rate (%)
Subject_Disorder	69.3
is_equivalent	80.4
Total	72.6

**Fig. 10:**
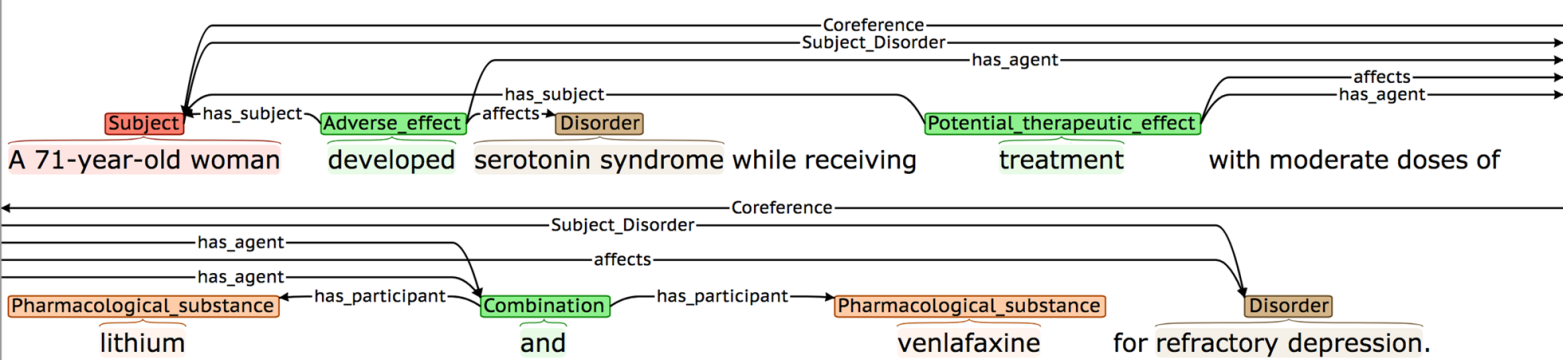
Complex sentence containing *Subject_Disorder* relation

*Subject_Disorder* relations were sometimes overlooked in more complex sentences, such as the one shown in Fig. 10, where the disorder (i.e., *refractory depression*) suffered by the subject at the time of the administration of the treatment occurs at the end of the sentence, after the introduction of several other types of complex and interlinked information.

**Fig. 11:**
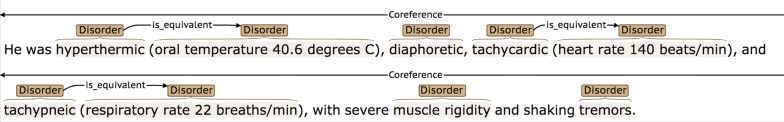
Incorrect *is_equivalent* annotation example

In certain cases, one of the annotators was found to be over-annotating *is_equivalent* relations. In Fig. 11, for example, the text spans in parentheses correspond to *descriptions* of the disorder names that precede the parentheses, rather than constituting alternative names for the disorders. Since these bracketed phrases do not comply with our guidelines for *Disorders,* they should NOT be annotated as such, and there should be no *is_equivalent* relations.

### Event attribute agreement rates

The agreement rates for the assignment of event attributes are shown in Table 12.

**Table 12:** Agreement rates for attribute assignment (F-Score)

Attribute type	Agreement rate (%)
Negated	75.6
Speculated	76.5
Manner	58.9
Total	70.9

For the *Negated* and *Speculated* attributes, comparably high levels of agreement are achieved. Since both of these are binary attributes (i.e., the value is either *true* or *false*), the decision is more straightforward than for the three-valued *Manner* attribute.

**Fig. 12:**
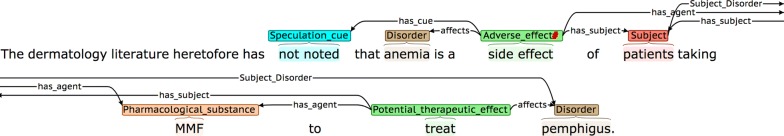
Speculated cue containing a negation word

Confusion may arise when a negation word occurs in the sentence, but it does not negate the event. In Fig. 12, for example, the negation concerns the lack of a previous *mention* of the event the literature, rather than saying it is not true. Since this phrasing suggests that the authors consider the event to be plausible, the assignment of the *Speculated* attribute seems more appropriate than the *Negation* attribute.

**Fig. 13:**
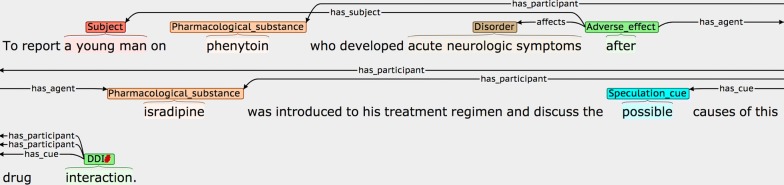
Incorrect assignment of a *Speculation_cue*

Similar care has to be taken to ensure that speculative phrases occurring in the sentence are indeed modifying the event of interest. One of the annotators marked the DDI event shown in Fig. 13 as *Speculated,* according to the presence of the word *possible.* However, the speculation is not related to *whether* the *DDI* took place, but rather about what *caused* it.

**Fig. 14:**
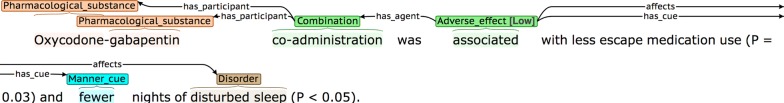
A complex case of assigning a *Low Manner* value

For the *Manner* attribute, agreement is usually reached when the manner cue directly modifies the event trigger. However, more careful reading is needed in sentences such as Fig. 14, where the description of the disorder *disturbed sleep,* rather than a modification of the event trigger, provides evidence that a *Manner* value of *Low* should be assigned to the AE event.

### Coreference agreement rates

Since coreference is annotated at the level of event participants, we calculate agreement for coreference annotation as follows:

- We examine events that are agreed by both annotators, according to the *relaxed* criterion for event matching introduced above.
- For the agreed events, we consider participants that have been annotated as *Coreferring_mention* by at least one of the annotators. A match is defined as a case in which the following conditions hold:
  - Both annotators have identified *Coreferring_mention* spans that overlap with each other AND
  - The *Coreferring_mention* spans are assigned the same semantic role in the matched events AND
  - The *Coreferring_mention* spans annotated by each annotator are linked to the same NE in a neighbouring sentence via a *Coreference* relation (i.e., the linked NEs must have overlapping spans, and must be assigned the same NE category).

**Fig. 15:**
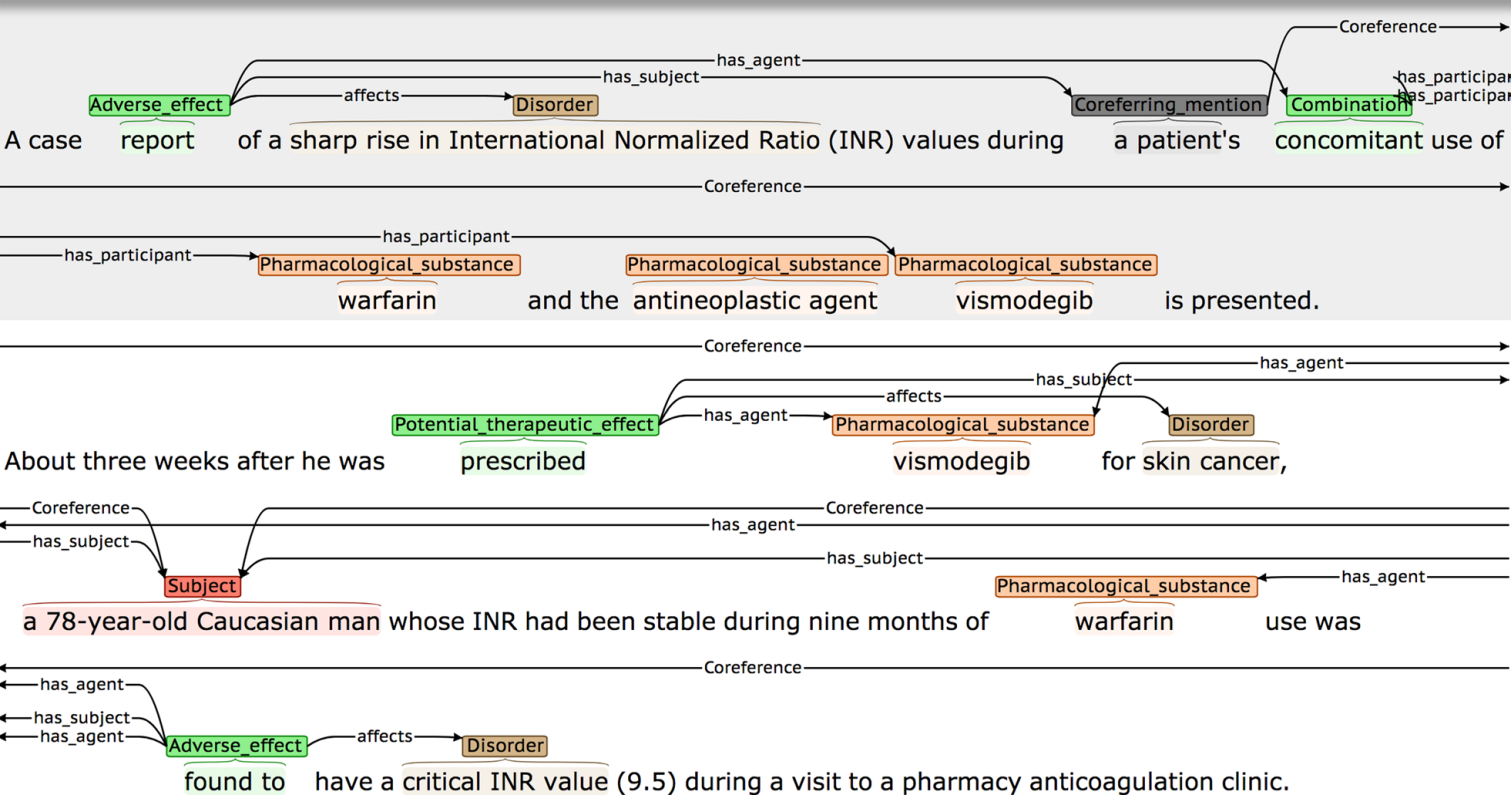
Example of *Coreferring_mention* appearing in sentence before linked NE

**Fig. 16:**
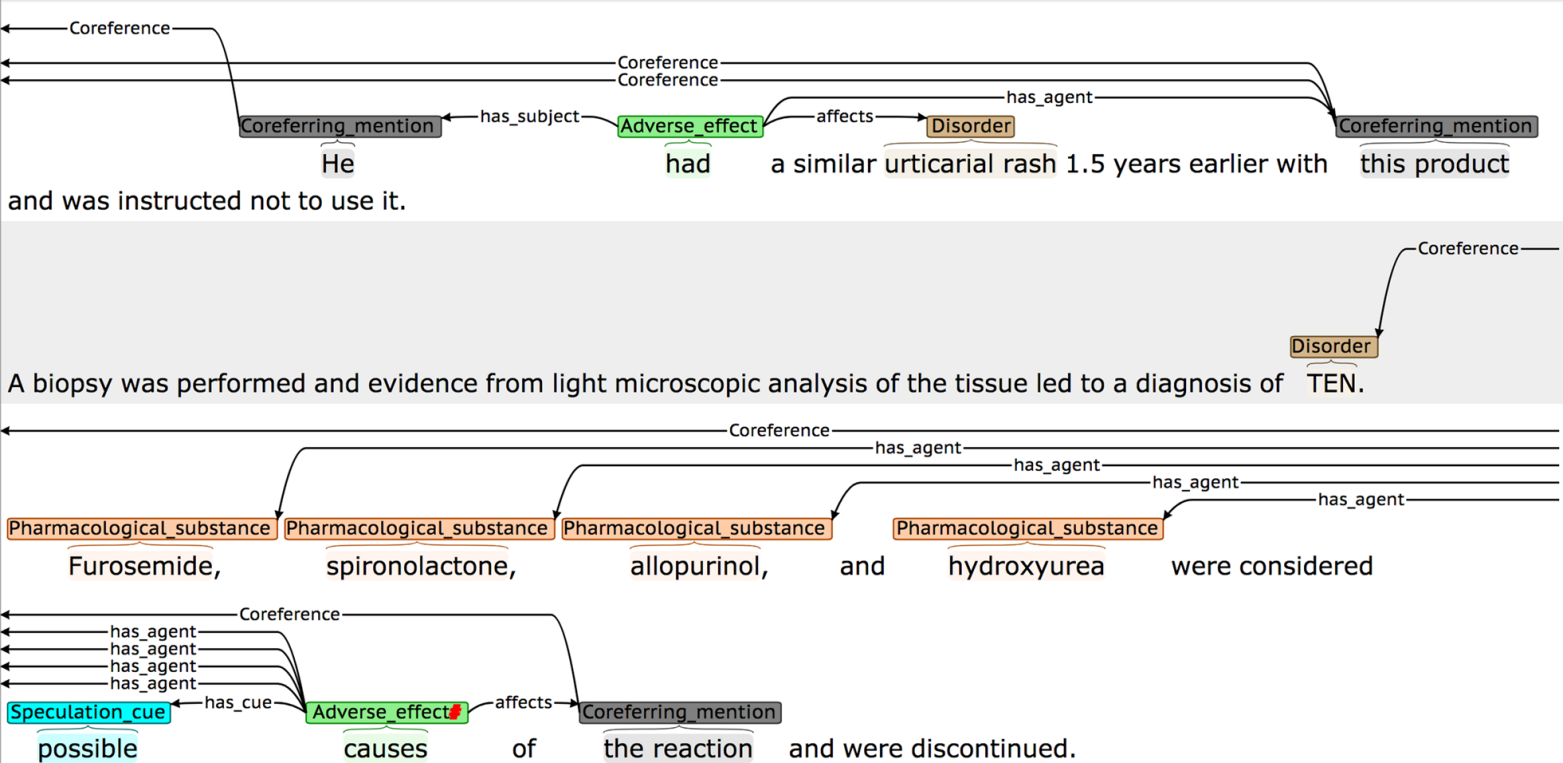
Example of potentially ambiguous *Coreferring_mention*

**Fig. 17:**
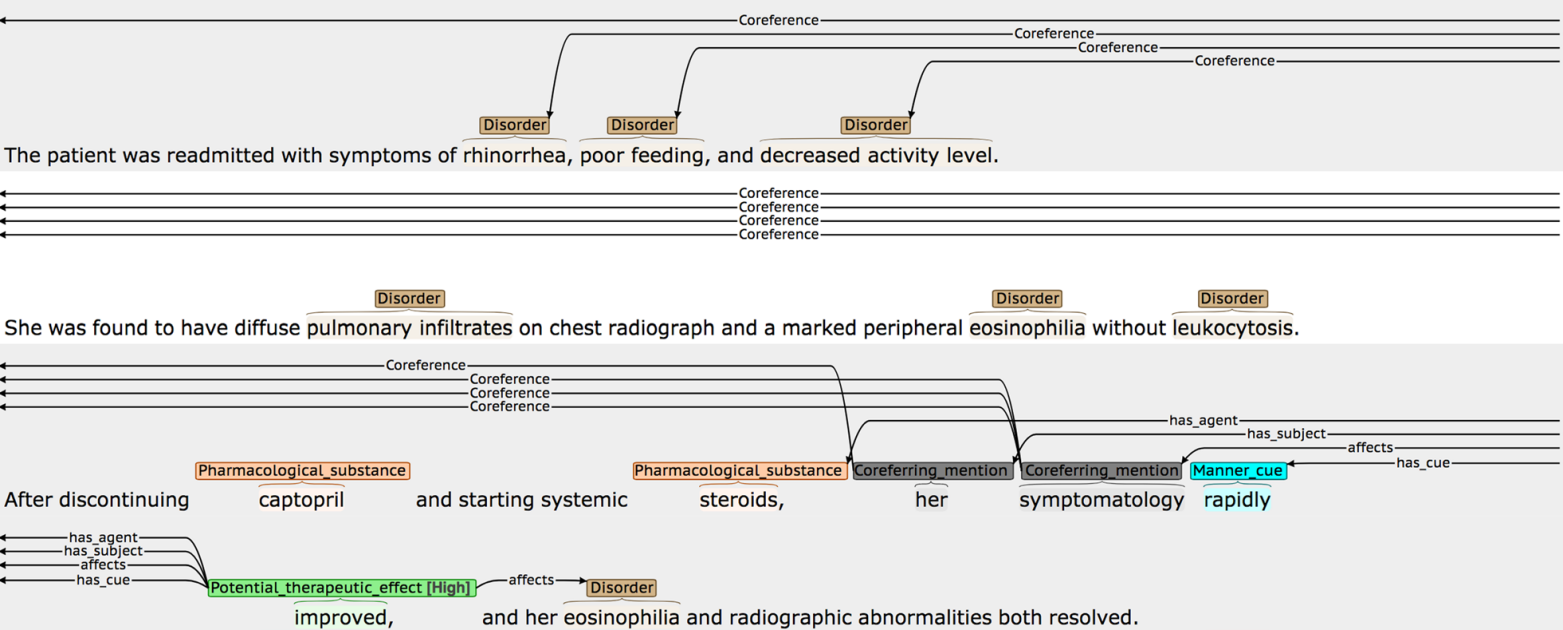
Example of larger distance between *Coreferring_mention* and linked NEs

By applying these criteria, we achieved an agreement rate of 50.91 F-Score. This is somewhat lower than the agreement rates for other levels of annotation. Although coreference involving *Subjects* is most common, and is generally straightforward (i.e., an initial descriptive phrase introducing the subject(s) will subsequently be referenced using phrases such as *she* or *them,* etc.), our IAA analysis showed that there were several types of more difficult cases that could lead to disagreements, some of which are illustrated in Figs. 15, 16 and 17.

Although the more descriptive NE phrase is normally introduced prior to the shorter co-referring phrases, this is not always the case, as shown in Fig. 15. Here, it is necessary to read forward to the second sentence to determine that the underspecified phrase *a patient* in the first sentence actually refers to *a 78*-*year*-*old Caucasian man.* It was found that one of the annotators initially tended only to look forwards for coreferring mention phrases, and so was often missing cases such as this. Examples such as the one shown in Fig. 16 can also cause problems, since it may be ambiguous whether the phrase *the reaction* in the final sentence refers to the disorder *TEN* or *urticarial rash.* Finally, in Fig. 17, it is necessary to read the text carefully to realise that the phrase *symptomology* refers back to symptoms introduced two sentences previously.

### Normalisation results

The HYPHEN method that we introduced in the *Methods* section can be readily adapted to normalise NEs of different semantic types to different terminological resources. Various readily-available, large-scale resources include concepts of the types that are covered by the *Pharmacological_substance* and *Disorder* NE types in PHAEDRA. Therefore, our decision of which terminological resource to use as the basis for normalising each of these concept types was driven by two main criteria:

1. To maximise the number of NEs that can be mapped to concept IDs, since this helps to ensure that the corpus annotations are as useful as possible.
2. To ensure that HYPHEN can achieve acceptable results when normalising NEs to the chosen resources.

We considered MeSH and DrugBank [3] as potential normalisation resources for *Pharmacological_substance* and MeSH and SNOMED-CT [74] as potential resources for *Disorder,* and we calculated the total number of NEs in PHAEDRA that could be mapped to each of these resources by HYPHEN. The results are shown in Table 13:

**Table 13:** Number of *Disorder* and *Pharmacological_substance* NEs that can be mapped by HYPHEN to different terminological resources

NE type	Target resource	Number of NEs normalised
Disorder	MeSH	2534
	SNOMED-CT	3094
Pharmacological_substance	MeSH	7156
	DrugBank	5651

For the final mappings, we chose the resources which allowed the greatest number of NEs to be mapped to concept IDs, i.e., UMLS concept unique identifiers (CUIs) associated with SNOMED-CT concepts for *Disorder* and MeSH IDs for *Pharmacological_ substance*. Both of these resources have constituted the target resources for normalising the respective entity types in other research efforts (e.g., [54, 110]), thus helping to demonstrate the utility of the methods.

In order to demonstrate that HYPHEN can maintain its high levels of performance when normalising NEs to these resources, we applied it to relevant gold standard annotated corpora that include mappings to our chosen resources, i.e., the ShaRE/CLEF corpus [54] (see Table 14) in which disorders are normalised to UMLS concept unique identifiers (CUIs) associated with SNOMED-CT concepts, and the Biocreative V CDR corpus [55] (see Table 15), in which chemicals are normalised to MESH IDs. In each case, we compare the performance of HYPHEN to two different baselines:

- Dictionary lookup (i.e., exact matching of NEs against variants in the respective resource).
- Results of applying the UMLS Norm program [116], which transforms terms in various ways, such as removing word inflections, stop words and re-ordering words alphabetically to try to match terms occurring in text against variants listed in a resource.

**Table 14:** Comparison of HYPHEN against baselines on the normalisation of disorder NEs in the ShARE/CLEF corpus

Method	Precision (%)	Recall (%)	F-score (%)
Dictionary lookup	90.34	59.51	71.76
UMLS Norm	84.82	66.65	74.65
HYPHEN	82.28	86.90	84.53

**Table 15:** Comparison of HYPHEN against baselines on the normalisation of chemicals in the BioCreative V CDR corpus

Method	Precision (%)	Recall (%)	F-score (%)
Dictionary lookup	98.07	78.78	87.36
UMLS Norm	96.11	82.82	88.97
HYPHEN	96.64	95.91	96.27

Tables 14 and 15 show that HYPHEN can considerably outperform the baselines in normalising both types of NEs. The performance for normalisation of disorders in the ShARE/CLEF corpus is only slightly lower than that reported in Table 6 for normalisation of diseases to MeSH concepts in the BioCreative V CDR corpus (87.03% F-score). This result helps to demonstrate the robustness and stability of HYPHEN when the parameters of the normalisation task change, since the ShARE/CLEF corpus concerns both a different text type (clinical records rather than biomedical abstracts) and normalisation to a different terminological resource, compared to the task reported in Table 6. Normalisation performance is even higher for chemicals, possibly because the types of names used to refer to them are frequently rather finite or follow rule-based conventions [117].

In Tables 16 and 17, we report on the number of *Disorder* and *Pharmacological_substance* NEs to which HYPHEN is able to assign IDs corresponding to concepts in either SNOMED-CT or MeSH. We compare this figure to the number of ID assignments achieved by applying the baseline methods, which is considerably lower.

**Table 16:** Number of disorder NEs in PHAEDRA normalised to SNOMED-CT concepts by HYPHEN, compared to baselines

Method	Total terms normalised	% total terms normalised
Dictionary lookup	2292	56.26
UMLS Normalised String lookup	2711	66.54
HYPHEN	3094	75.94

**Table 17:** Number of pharmacological substance NEs in PHAEDRA normalised to MeSH concepts by HYPHEN, compared to baselines

Method	Total terms normalised	% total terms normalised
Dictionary lookup	6064	74.88
UMLS Normalised String lookup	6562	81.03
HYPHEN	7156	88.37

Table 18 shows examples of how HYPHEN’s pipeline of variant generation techniques can help to achieve successful normalisations of NEs in PHAEDRA. In cases where multiple transformations of the original NE are required to achieve a match with a variant listed in the resource, the different steps and intermediate variants generated are shown.

**Table 18:** Examples of successful normalisations determined by HYPHEN in PHAEDRA

Original Term	Category	Transformations		Concept ID assigned
		Technique	Variant generated	
*supratherapeutic INRs*	Disorder	(1) Acronym disambiguation	*supratherapeutic international normalized ratios*	UMLS: C0853225
		(2) Plural to singular	*supratherapeutic international normalized ratio*	
*cutaneous pigmentation*	Disorder	Neoclassical-English	*skin pigmentation*	UMLS: C1269684
*liver toxicity*	Disorder	English-Neoclassical	*hepatic toxicity*	UMLS: C0348754
*abnormalities in liver function tests*	Disorder	Syntactic variant generation	*abnormal liver function tests*	UMLS: C0151766
*immunosuppression*	Pharmacological substance	Syntactic variant generation	*immunosuppressant*	MeSH: D007166
*antihistaminics*	Pharmacological substance	Synonym generation	*antihistamines*	MeSH: D006633
*convulsive seizures*	Disorder	(1) Plural to singular	*convulsive seizure*	UMLS: C0036572
		(2) Syntactic variant generation	*convulsion seizure*	
		(3) Synonym generation	*fit-convulsion*	

### Final corpus analysis

In this section, we provide and discuss some characteristics of the final PHAEDRA corpus, which help to demonstrate that it will be usable as intended, i.e., to allow the training of ML tools to detect various types/levels of information relating to PV in textual data. Table 19 shows general statistics for the different annotation levels, while Table 20 shows some more detailed statistics regarding event participants and attributes.

**Table 19:** Final corpus statistics

Annotation level	Annotation type	No of anns. (discontinuous)	Av. words per ann.	Unique spans, triggers or cues	Total anns
NEs	Pharmacological_substance	8099 (38)	1.22	1853	13,726
	Disorder	4075 (63)	1.90	1998	
	Subject	1552 (30)	2.03	712	
Events	Adverse_effect (AE)	1375	1.15	309	3166
	Potential_therapeutic_effect (PTE)	893	1.06	195	
	Combination	560	1.05	81	
	DDI	338	1.02	12	
Event Participants	has_agent	2424			7523
	Affects	2308			
	has_subject	909			
	has_participant	1882			
Event Attributes	Negated	61		19	604
	Speculated	448		117	
	Manner	95		47	
Coreference		212			212
Relations	Subject_Disorder	636			941
	is_equivalent	305

**Table 20:** Statistics about event participants and attributes

Event statistic	Count
At least one *has_subject* participant	890
At least one interpretative attribute	583
Three participant types	564
*has_agent* participant is an event	415

The numbers of NEs, events and relations are similar to those that have previously been shown to be sufficient for ML training purposes (e.g., [39, 42, 43, 61, 85]). This is confirmed in the next section, which reports on the use of the corpus for NE detection and event extraction.

The number of events annotated per abstract varies between 0 and 20, with 75 abstracts containing no events at all. Thus, it is not always the case that abstracts containing the appropriate entity types, and which our search strategy selected as covering the relevant subject, actually contain events. Event recognition tools should be capable of distinguishing between cases of valid and invalid events, when the correct NE types are present in a sentence and the right subject area is covered by a document. The fact that PHAEDRA contains abstracts with varying numbers of events should help to ensure that event recognition models trained using the corpus can learn the contexts in which entities of the correct type should be linked to form an event, and thus avoid the potential problem of “over-trained” models, i.e., those that are liable to recognise events, even when they are not actually present.

There is a large difference in the range of unique triggers annotated for each event type, with the greatest variety of triggers occurring for the AE event type. Common triggers for this event type include those denoting causality, association or risk (*induced, associated, caused, resulting, related, risk*) or temporal terms (*after, follow, during),* while others relate to the commencement of a disorder (*develop, occur, trigger, producing, manifestation*), to specific effects on a disorder (*exacerbation, aggravating, worsening, acceleration),* to harmful effects (*toxic, side effect, intoxication, harmful effects, neurotoxicity*) or to potential for harm (*borne in mind, raises important questions, prescribed with caution*).

Triggers for PTE events appear to have a less variable semantic scope than AE events, consisting mainly of words relating to treatment, therapy or administration (*treated, received, prescribed, therapy, taking, use)* or to the positive effects of treatments (*effective, prevent, resolve, efficacy, improved, controlled, decreased, benefit, recovery, disappeared, reversing*).

There are also many different triggers for *Combination* events (*and, with, plus, combination, concomitant, concurrent, co*-*treatment, conjunction, simultaneously, mixtures, multiagent, given with, used together, added, boosted*, etc.). In contrast, the triggers for interactions are highly restricted, mostly consisting of variations on the word *interaction* (*interactions, interact, interacts*), possibly with a modifier (*pharmacokinetic interaction, drug interaction*).

Negation cues are also very limited and include *no, not, without, failed, despite* and *inactive*. The most common cues for *High* and *Low Manner* cues, respectively, are *significant* and *rare.* Other cues mainly relate to severity (*severe, drastically, critically, dangerously, life threatening, minor*), completeness of the effect (*completely, partially*) or frequency (s*eldom, uncommon, less frequently*). Cues for *Speculated* events are far more variable. The most common are *potential, may, possible, risk* and *probable,* while others mark events that are mentioned in an evaluative context (*assess, evaluate, investigate, study, examine, hypothesise*), various degrees of tentativeness (*suggest, seem, believe, think, propose, presume, unlikely*) or expressions of caution, which may indicate *potentially* harmful effects (*with caution, care should be taken/exercised, close attention must be paid).*

In the GENIA event corpus [118], it was found that approximately 8% of all the molecular-level events were marked for speculation, which is around half as many as in the PHAEDRA corpus (14%). The higher level of speculation in our corpus could be due to the need to report drug effects with some degree of caution, since different patients are likely to respond/react in different ways. The occurrence of this type of speculation is reinforced by some of the most common types of speculation cues in our corpus (e.g., *potential* and *risk),* which do not feature at all in the list of the most common speculation cues in the GENIA event corpus.

One of our motivations for using event annotations is to link contextual information about medical subjects with drug effects. The statistics for NE annotation show both that such information is frequently specified in abstracts, and that there is a large degree of variation in the nature of subject descriptions. These variations may concern numbers in a subject group, age, gender, life stage (e.g., *children adults, elderly),* ethnicity/nationality, lifestyle habits, (*a white man with a long history of smokeless tobacco use*), genetic characteristics (*subjects with the cyp2c19 poor metabolizer genotypes*) or previous surgery (*an 18*-*yr*-*old female kidney transplant recipient*), among others. The fact that around 28% of all events have at least one *has_subject* participant provides verification of the relatively high frequency with which drug effects are qualified with subject information. Furthermore, a quarter of all AE and PTE events identifies three distinct types of participants (i.e., *has_agent, affects* and *has_subject*), which clearly illustrates the need to move beyond binary relations to fully encode the different types of information about drug effects that are frequently specified in text.

A further motivation for using events is to allow complex causes of drug effects to be encoded. The fact that 415 events (around a fifth of all PTE and AE events) have *has_agent* participants that are themselves events (i.e., *Combination* or *DDI*) provides evidence that such descriptions occur with reasonable regularity.

As mentioned previously, events in our corpus are restricted to those whose triggers and participants all fall within the scope of a single sentence. This decision was mainly motivated by our desire to ensure that PHEADRA can be readily used to train practical TM tools, since most existing ML-based event extraction systems (e.g., [89, 91, 92]) are only designed to extract sentence-level events.

However, information in text can be described in many different ways, and the details of an event may not always fall neatly within the scope of a single sentence. Our co-reference annotation is intended to help with this, in that it is possible to annotate event participants that correspond to anaphoric expressions (which would normally be considered too vague to annotate as NEs), and to link them to their referent NEs in other sentences.

To ensure that our annotation scheme can sufficiently capture all relevant information about events that is specified in the text, we have manually analysed a small subset of PHAEDRA (20 abstracts) to identify the following types of cases:

- An event annotation is missing, incorrect or incomplete, according to the way in which the information is phrased in the text
- The annotation of the event and/or one or more participants is only possible according to our use of co-reference annotation

**Fig. 18:**
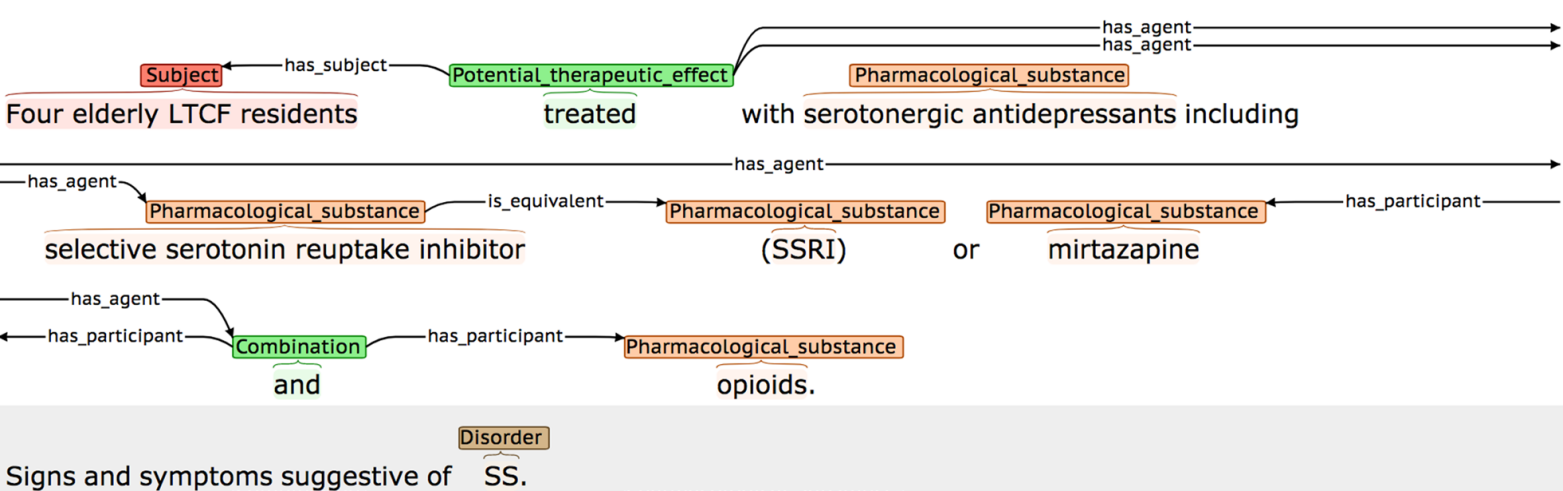
Missed event due to implicit reference to participant(s)

More specifically, we counted instances of five different cases in which event information extends beyond the boundaries of a single sentence, each of which is introduced below with an example:

1. Event is not annotated due to implicit reference to required participants (see Fig. 18).

In Fig. 18, it is implicit that the disorder *SS* (serotonin syndrome) mentioned the second sentence occurs as a result of the treatments mentioned in the first sentence (i.e., there is an implicit AE event). However, since the second sentence mentions neither the pharmacological substances nor anaphoric expressions that refer to them, no AE event is annotated.

**Fig. 19:**
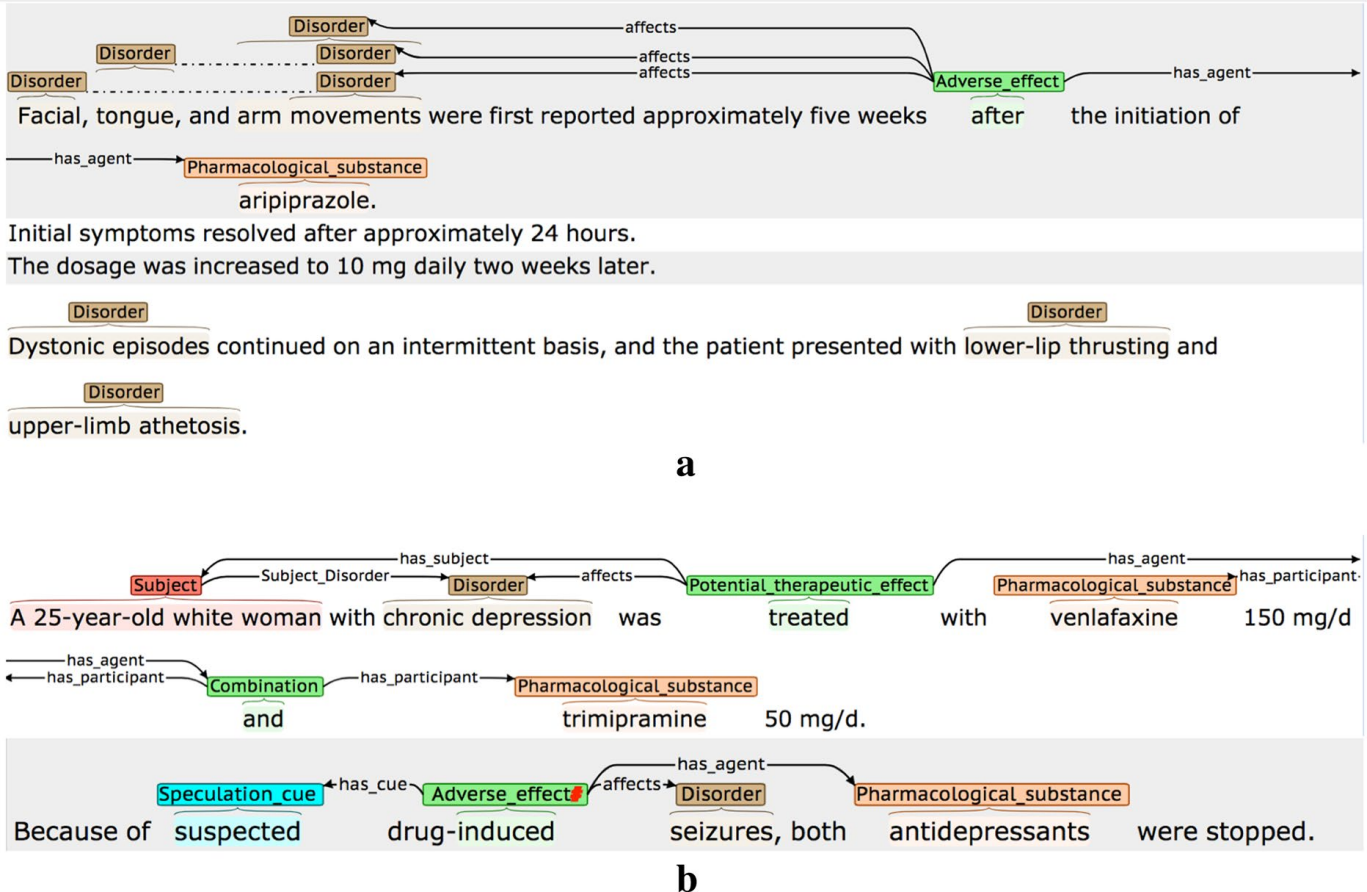
Event participants missed due to implicit reference. Two separate examples are shown (**a** and **b**); these are explained in the main text

(2) Events are annotated, but certain participants are missed due to implicit reference (see Fig. 19).

In Fig. 19a, it is implicitly understood that the disorders in the last sentence are caused by *aripiprazole*, which is mentioned only in the first sentence. Hence, the final sentence contains additional *affects* participants that cannot be annotated. Similarly, in the sentence shown in Fig. 19b, the second sentence is talking about the same case as the first, and hence it is implicit that the same medical subject in involved, although the phrasing means that this information cannot be annotated.

**Fig. 20:**
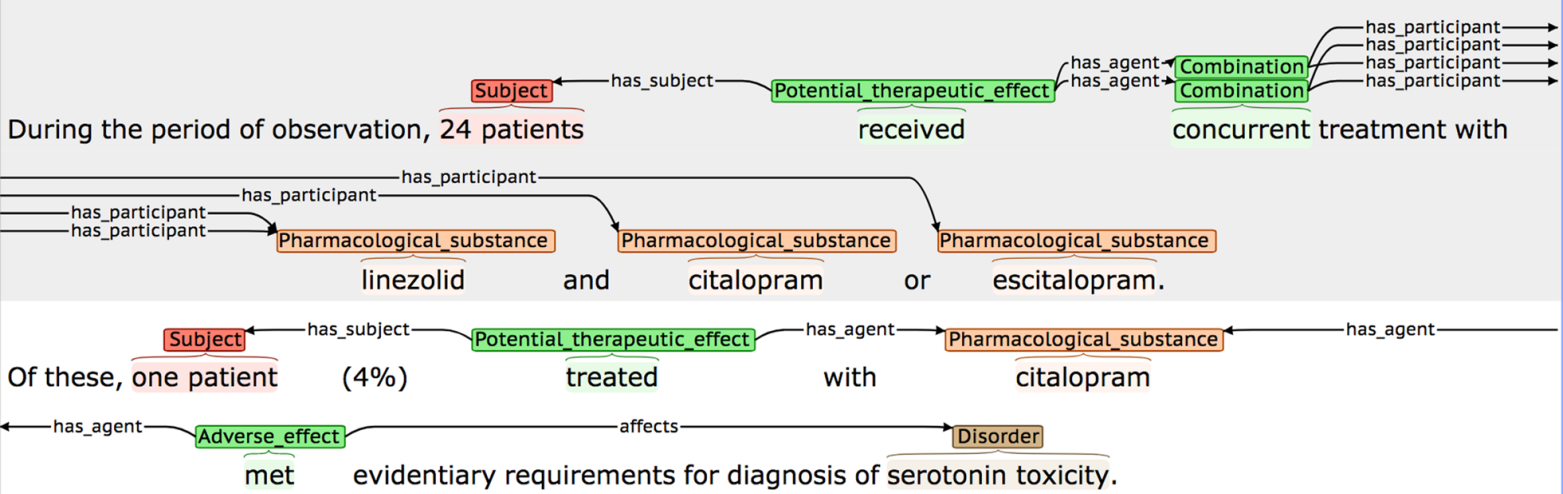
Event participants incorrect due to implicit reference

(3) Events are annotated, but implicit reference means that one or more participants are incomplete/incorrect (see Fig. 20).

In Fig. 20, the first sentence states that medical subjects received two different combinations of drugs (i.e., linezolid and citalopram *or* linezolid and escitalopram). However, although the second sentence explicitly states that an adverse reaction occurred in a patient treated with citalopram, it is implicitly understood that this actually means that the patient was treated with a *combination* of citalopram *and* linezolid. This leads to the *has_agent* participant being annotated incorrectly, even though the annotation seems correct when considering the second sentence in isolation.

(4) Co-reference annotation facilitates annotation of certain event participants.

**Fig. 21:**
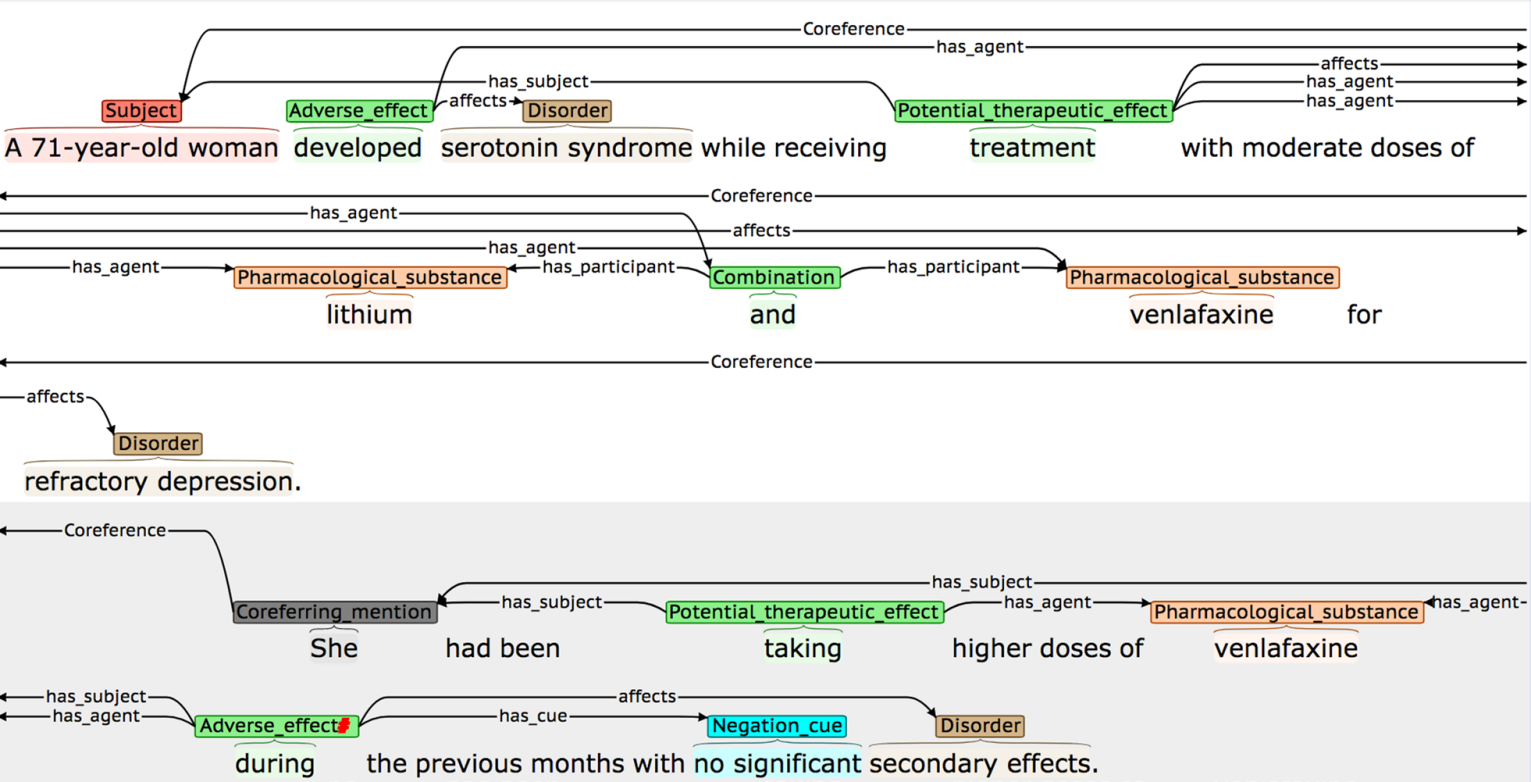
Co-reference allows identification of an event participant in another sentence

In Fig. 21, the co-reference annotation allows the information about the medical subject introduced in the first sentence (i.e., *A 71*-*year*-*old woman*) to be linked to both the PTE and the AE event in the second sentence, via the anaphoric expression *she*.

(5) Co-reference annotation allows annotation of events that would otherwise remain un-annotated.

**Fig. 22:**
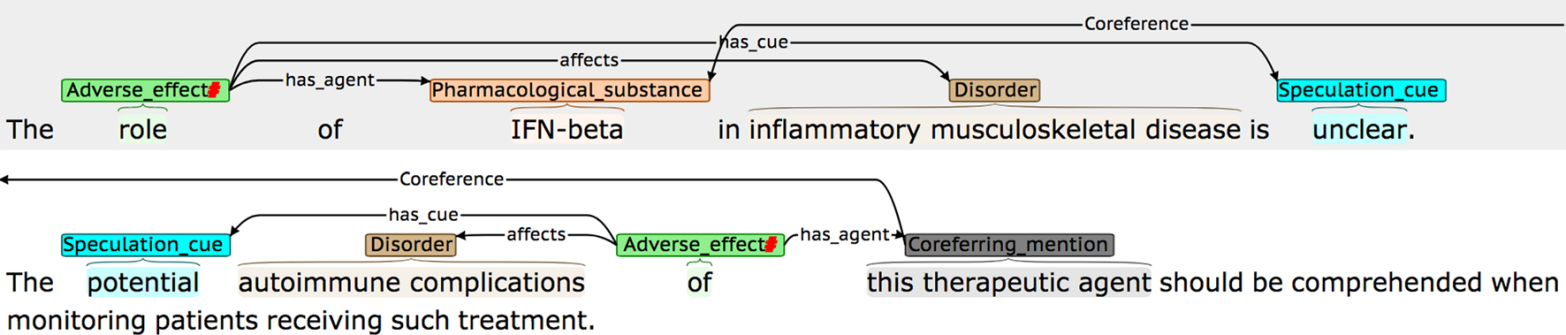
Co-reference facilitates annotation of event

In the second sentence of Fig. 22, the AE event denoting that *IFN*-*beta* (referred to in this sentence using *this therapeutic agent*) has the potential to cause autoimmune complications, would remain un-annotated, without the use of co-reference annotation.

The counts for each of the five cases are shown in Table 21, along with the total number of events in our 20 abstract sample.

**Table 21:** Counts of different cases of cross-sentence event information

Total events	278
Events unannotated	18
Events with missed participants	26
Events with incorrect participants	5
Events ONLY annotated due to coreference	3
Events with participants ONLY annotated due to coreference	16

An important result from our sample analysis is that it provides evidence that our annotation scheme is sufficient to capture the majority of information about events that is specified in text. In particular, of the 278 events that *are* annotated, 234 (i.e., 84.17%) can capture all information that is specified about the event. While co-reference annotation is only critical in a small number of cases to ensure that events are not missed, it helps with the identification of certain participants in a larger number of cases, thus helping to demonstrate its ability to ensure that event annotations are as complete as possible.

A further important result of our analysis is that the number of events that remain completely un-annotated in our sample, according to implicit references, is also quite small (i.e., 18). Thus, even if we had annotated events whose participants are described beyond the boundaries of a single sentence, then we may estimate an increase in the number of events annotated of only about 6.5%.

**Fig. 23:**
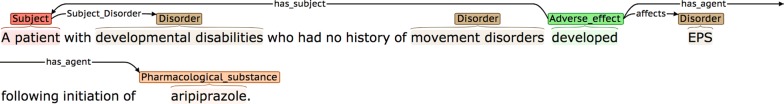
Conclusion sentence mentioning all event participants

Most of the issues with events that *are* annotated concern the lack of a phrase (either NE or anaphoric expression) that refers to the participant in the event-containing sentence. However, it is often the case that, although information may be scattered amongst sentences when it is initially introduced, the conclusion of the abstract tends to provide a concise summary of the main findings of the study, including all relevant participants. Such a sentence is illustrated in Fig. 23, where the medical subject is explicitly mentioned and can be linked to the AE and PTE events, even though the subject is not explicitly referred to in earlier sentences that introduce these events.

Our sample analysis suggests that the proportion of events that is completely missed due to implicit reference is quite small, and that events with missed participants are often stated more fully elsewhere in the abstract. As such, the benefits of sentence-based event annotation, in terms of the ready availability of a range of tools that can be trained to recognise them, outweigh the fact that a relatively small proportion of information is missed.

### Training of machine learning based text mining tools using the corpus

In this section, we demonstrate the utility of the final annotated PHAEDRA corpus, by reporting on its use to train two ML models (one for NE recognition and one for event extraction), employing commonly used tools.

### Automatic NE recognition

For NE recognition, we use NERSuite [119], which has previously been used to train other high-performance, medically-relevant NE tools [42, 120].

We randomly split the complete corpus into a training set (359 abstracts), development set (120 abstracts) and test set (118 abstracts). We trained an NERSuite model using the training set, and performed evaluation against the test set. The results are shown in Table 22, where we show both exact and relaxed span matching rates.

**Table 22:** NERSuite evaluation results

Category	Exact span match			Relaxed span match		
	P	R	F	P	R	F
Pharmacological_substance	89.2	64.5	74.9	94.4	69.9	80.3
Disorder	77.9	57.7	66.3	92.0	71.2	80.2
Subject	83.6	59.3	69.4	89.3	63.1	73.9
Total	85.7	62.2	72.1	93.2	69.3	79.5

Precision is high for all categories, and especially when the relaxed matching criterion is used. The lowest discrepancy between exact and relaxed matching rates occurs for the *Pharmacological_substance* category, which can be explained partly according to our previous observation regarding the lesser variability in the structure of these terms, compared to others, and partly since NEs belonging to this category are typically shorter than those belonging to the other categories (see Table 19), with many instances being only one word long. However, the large number of unique drug names in the corpus appears to contribute to the lower recall. Thus, the use of additional features from a domain specific drug resource is likely to be beneficial.

Recognition errors for the *Disorder* category are particularly likely to occur for longer spans (e.g., *angiomatous enlargement of the gingiva, Advanced Previously Untreated Non*-*Small*-*Cell Lung Tumors*) and spans that constitute or contain acronyms (e.g., *NSCLC, elevated INR*). The fairly large discrepancy between relaxed and exact matching rates for this category appears to occur largely for mentions of specific, descriptive disorders. In such cases, it is more likely that the model will predict a shorter, more general span that misses the descriptive details of the gold standard span, e.g., *swelling* instead of *swelling in his right buttock,* or *acute myelogenous leukemia* instead of *adult* *de novo* *acute myelogenous leukemia.* In terms of *Subject* annotations, recognition failures can occur for rare types of subjects, e.g., non-human subjects such as *mice, animals, Schistosoma mansoni,* or more unusual types of descriptions, such as *extensive metabolizers*. Fine details in long subject descriptions may also be missed, e.g., in one case, *adult patients* is predicted instead of *adult patients (15*-*60* *years old)*, while in another, *male* is predicted instead of *male 60 yrs of age.*

Given the related subject area, we can compare our results broadly to those reported for medically related NEs in the historical medical corpus, HIMERA, which also used NERSuite [42]. Using the default NERSuite configuration, the results achieved for the *Condition* NE class in HIMERA (broadly comparable to our *Disorder* class) were 73%/82% F-Score for exact/relaxed span matches, which are quite similar to our scores of 66%/80% F-score. It should also be noted that our current corpus covers a wider range of disorders than HIMERA, which was primarily restricted to documents concerned with lung diseases. Although higher scores for disorder recognition were achieved in another study [120], i.e., 75% exact/88% relaxed F-Score, additional dictionary features and a larger training corpus were used; it was shown in experiments on the HIMERA corpus that the use of dictionary features can boost F-Scores by up to 6%.

Our recognition performance for *Subject* entities is somewhat lower than that achieved for the HIMERA corpus (74% relaxed/81% exact for HIMERA vs. 69% relaxed/74% exact for our corpus). However, a large number of the subject annotations in HIMERA are short, vague spans such as *cases* and *patients*, whilst we require longer and more descriptive subject phrases to be annotated. HIMERA also annotated treatments, although they had wider semantic scope than our pharmacological substances, covering all types of treatment and investigational techniques. This helps to explain why our performance for pharmacological substances (75% exact/80% relaxed F-Score) is higher than that achieved in HIMERA (64% relaxed/57% exact F-score).

### Automatic event recognition

We used the EventMine system [88] for automatic recognition of events. Regular improvements to this system have assured its state-of the-art performance when applied to texts belonging to different subject areas and text types [90, 91, 104, 121]. EventMine applies a pipeline of TM tools to text in which NEs have already been recognised, in order to recognise event triggers and participants, to assign appropriate semantic categories to them and to link them together into potentially complex event structures.

**Fig. 24:**
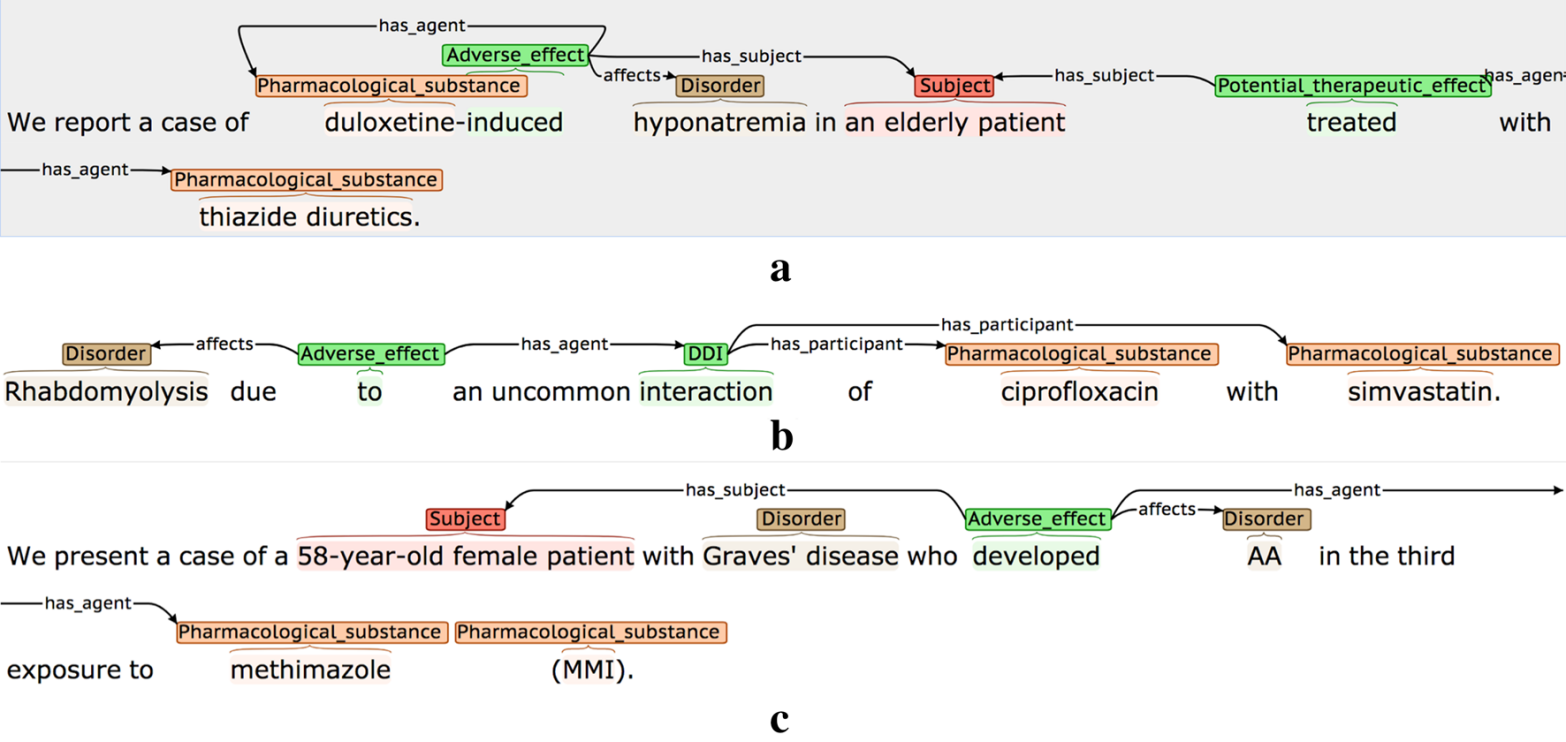
Examples of events successfully detected by EventMine. The three different examples (**a**, **b** and **c**) are discussed in the main text

We trained an EventMine model using 479 annotated abstracts (from the training and development sets), and used the remaining 118 abstracts (in the test set) for evaluation. Figure 24 shows examples of complex events that are successfully detected by the model, including those with three different types of participants (a), and those whose participants are themselves events (b). Sentence (c) shows that the model can successfully distinguish the correct event participants, even when there are multiple NEs of the same type in the sentence.

To assess of the performance of the model, we firstly determined how well it can identify the same events that are annotated in the gold standard, by matching triggers (see Table 23). We used the same relaxed criteria for matching triggers that were introduced above for calculating IAA, according to our observations about the diversity of potential triggers for most of our event types, and the fact that there may be multiple candidate triggers for a particular event within a given sentence.

**Table 23:** EventMine results in identifying *and* classifying event triggers

Event type	P (%)	R (%)	F (%)
Combination	61.5	35.6	45.1
DDI	66.4	79.8	72.5
Potential_therapeutic_effect	67.9	43.4	53.0
Adverse_effect	79.9	60.3	68.8
Total	71.6	54.1	61.6

**Fig. 25:**
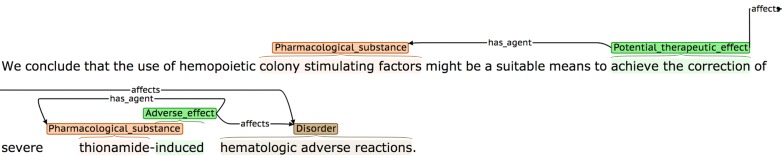
Unusual phrasing of a PTE event NOT recognised by EventMine

**Fig. 26:**
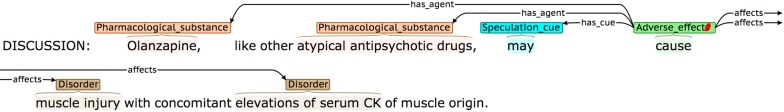
AE event with unexpected sentence structure NOT detected by EventMine

The imbalance between precision and recall appears to be a general feature of models trained using EventMine. However, recall is very high for DDI events, probably due to the smaller number of triggers that are possible for this event type. Acceptable levels of precision are obtained for all event types; the highest precision is achieved for *Adverse_Effect* events, providing evidence that certain words and phrases in a sentence can reliably predict the presence of this event type. Lower recall may be caused by events with unusual phrasing (see Fig. 25), or unexpected sentence structure (see Fig. 26) being missed by the model.

**Fig. 27:**
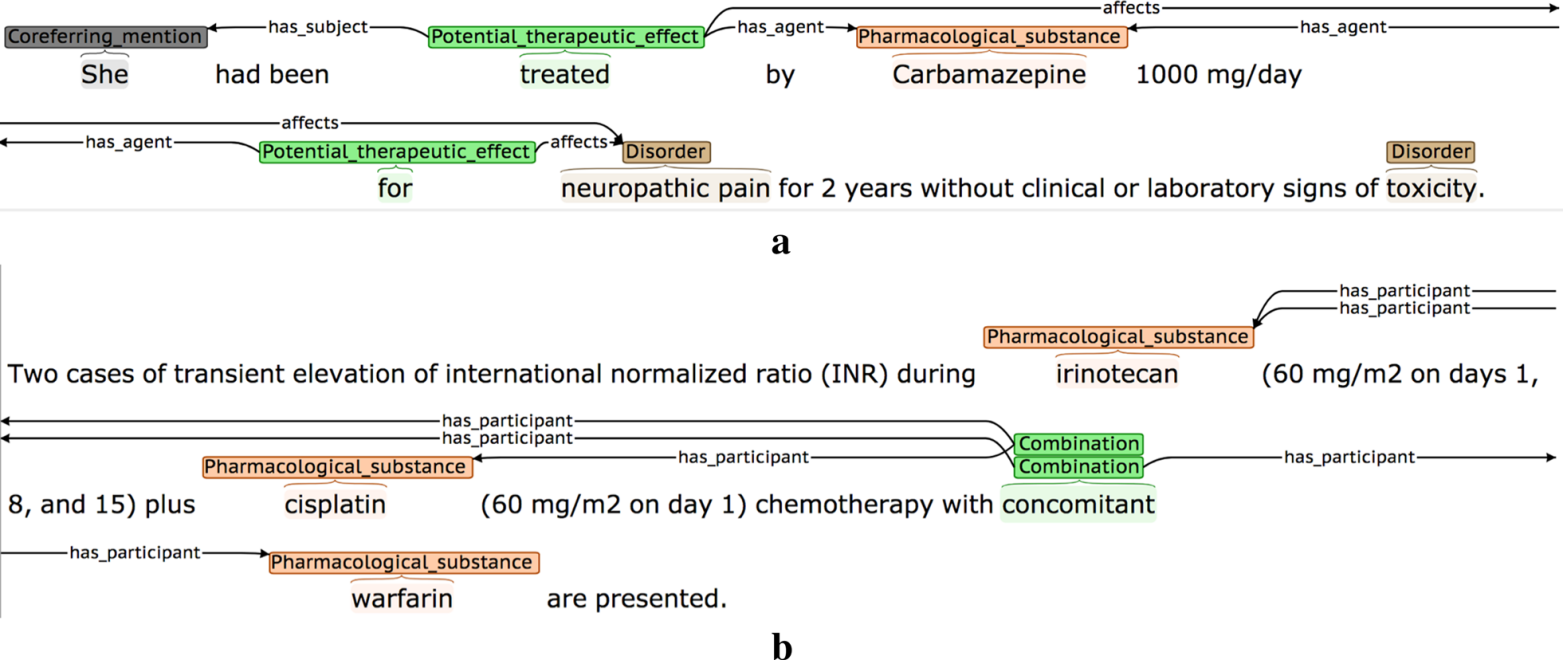
Incorrect events detected by EventMine. Two separate examples (**a** and **b**) are illustrated; these are described in the main text

Figure 27 shows some examples of events erroneously recognised by EventMine. In sentence (a), only a single PTE event should have been recognised. However, presumably since *treated* and *for* are both common triggers for *PTE* events, two separate events were recognised by the model. In sentence (b), the gold standard includes only a single *Combination* event, with all three pharmaceutical substances being identified as participants. However, presumably because *Combination* events occur more commonly with only two participants in the training data, EventMine has recognised two separate events with the same trigger, but each linking a different pair of drugs.

Table 24 reports on the degree to which event participants of different types are correctly recognised in *matched* events, i.e., those events recognised by EventMine whose triggers can be matched to events in the gold standard.

**Table 24:** EventMine results in identifying event participants

Role type	P (%)	R (%)	F (%)
Affects	90.2	81.2	85.4
has_agent	82.4	81.2	82.1
has_subject	83.2	83.2	83.2
has_participant	93.4	90.6	92.0
Total	87.9	84.5	86.1

In general, the results for all participant types are quite high, with the best results being achieved for the *has_participant* role. The results provide evidence that the EventMine model is able to identify both core participants and contextual information about medical subjects with a high degree of accuracy.

We also carried out an evaluation based on the one used in the BioNLP ST evaluations. The original evaluations determined the extent to which models are able to carry out the joint task of identifying the existence of events *and* detecting their primary participants, i.e., their *Themes* (the participants undergoing change) and their *Causes*. To perform a similar evaluation, we consider the primary participants of our event types to be *has_agent* and *affects* for the *AE* and *PTE* event types, and *has_participant* for the *DDI* and *Combination* event types. While in the BioNLP STs, automatically recognised events are only matched against those in the gold standard if they have overlapping trigger phrases, we use the more relaxed criteria for matching trigger phrases, as introduced above. The results are shown in Table 25.

**Table 25:** EventMine results in identifying events *and* their primary participants

Event type	P (%)	R (%)	F (%)
Combination	56.5	28.9	38.2
DDI	61.9	65.7	63.7
Potential_therapeutic_effect	55.6	25.7	35.2
Adverse_effect	72.0	39.0	50.6
Total	63.6	37.5	47.2

The overall results are slightly lower than when EventMine was applied to a task of broadly comparable complexity and text type, i.e., the BioNLP’09 ST of extracting molecular level events from biomedical abstracts [81], for which an overall F-Score of 53.29% was achieved [88]. In the corpus used for this ST, however, around a third of all events have only single participant, and performance for these event types was quite high (70.44% F-Score). In contrast, all events in our corpus can potentially have multiple participants and in most cases, multiple participants are actually required. As such, we would expect overall event extraction performance to be lower for our corpus.

EventMine’s performance for events of comparable complexity is broadly similar across the two corpora. For example, in the BioNLP shared task, the *Binding* event type is comparable to our *Combination* and *DDI* event types, in that there are potentially multiple primary participants, each having the same semantic role label. EventMine’s performance in recognising *Binding* events was 52.62% F-Score, while the combined performance for *Combination* and *Binding* events in our corpus is 50.98%. Similarly, performance on the BioNLP’09 corpus for event types that take two types of primary participants (i.e., *Theme* and *Cause*) is 40.60% F-score, while for our two event types that have two primary participant types (i.e., *AE* and *PTE*), the performance is 44.96% F-score.

The parallels between the performance of EventMine on events of similar complexity but differing semantics provide additional evidence that the annotation quality in the PHAEDRA corpus is similar to that in the widely used BioNLP’09 ST corpus, which has been used extensively to train event extraction models.

## Conclusions

In this article, we have reported on the development of a novel, freely available corpus, PHAEDRA, that is annotated with multiple levels of information that allow complex details relevant to PV studies to be encoded. It is intended that this will act as a stimulus for the development of TM methods that are considerably more sophisticated than those currently available for this domain, which are at present largely restricted to identifying binary relations between drugs and diseases or drug–drug interactions. In particular, the event-centric annotations in our corpus can encode drug effects with complex, multi-drug causes, and include detailed information about the medical subjects in which such effects occur. Moreover, interpretative attributes categorise drug effects according to their intensity, and can distinguish between tentative and speculated effects. The automatically-added links between *Disorder* and *Pharmacological_substance* NEs add further value to the corpus, e.g., by helping to group together events from different documents that involve the same concepts, even if these concepts are mentioned in different ways.

Despite the complexity of the annotations, their automated recognition is within the capabilities of current TM tools. We have provided an initial demonstration of this by training baseline classifiers for two of the levels of annotated information, i.e., NEs and events, using existing tools that have been widely applied to other types of annotated corpora. Even by using the default settings and feature sets in training these models, we obtained performance levels that compare highly favourably to those achieved for related corpora. We hope that our work, including our analysis of the types of errors made by the classifiers, will encourage the development of novel classifiers with even higher performance.

As future work, we intend to extend our corpus in several ways. Firstly, we aim to extend/improve upon the current annotations in the corpus. This will include manual verification/editing of the automatically-added “silver-standard” links between NEs and concept IDs, as well as the addition of further relation/event types, e.g., to encode pharmacokinetic mechanisms responsible for drug effects. We also plan to augment the documents in the corpus with further types of text that have been identified as being highly relevant to PV studies, including clinical records and social media postings. Our ultimate aim is to facilitate the training of ML classifiers that can robustly extract and combine complex information from multiple information sources. By mapping this information to an upper-level ontology (e.g., [122], which includes a set of relations to describe processes in terms of their participants and their actions, as well as the ability to encode attributes of such processes), we can increase the ease with which information extracted by these classifiers can be queried and integrated with domain-specific knowledge from other information sources. These planned improvements will all help to improve the feasibility of developing novel, high-performance tools that can assist curators in creating and maintaining PV knowledge resources that are as comprehensive and complete as possible, in order to ensure that such resources constitute a reliable and trustworthy means of assessing drug safety in patients with diverse characteristics.

## Abbreviations

ADE: adverse drug effect
AE: adverse effect
DDI: drug–drug interaction
IAA: inter-annotator agreement
ML: machine learning
NE: named entity
PTE: potential therapeutic effect
PV: pharmacovilgilance
TM: text mining
ST: shared task
